# Human–Animal Interaction and Perinatal Mental Health: A Narrative Review of Selected Literature and Call for Research

**DOI:** 10.3390/ijerph181910114

**Published:** 2021-09-26

**Authors:** Shelby E. McDonald, Camie A. Tomlinson, Jennifer W. Applebaum, Sara W. Moyer, Samantha M. Brown, Sue Carter, Patricia A. Kinser

**Affiliations:** 1 Children, Families, and Animals Research (CFAR) Group, LLC, Richmond, VA 23223, USA; 2 School of Social Work, Virginia Commonwealth University, Richmond, VA 23284, USA; 3 Department of Sociology and Criminology & Law, University of Florida, Gainesville, FL 32611, USA; jennyapplebaum@ufl.edu; 4 School of Nursing, Virginia Commonwealth University, Richmond, VA 23298, USA; moyersw@vcu.edu (S.W.M.); kinserpa@vcu.edu (P.A.K.); 5 School of Social Work, Colorado State University, Fort Collins, CO 80523, USA; samantha.brown@colostate.edu; 6 The Kinsey Institute, Indiana University, Bloomington, IN 47405, USA; suecarter@indiana.edu

**Keywords:** perinatal, human–animal interaction, pets, mental health, biomarkers

## Abstract

There is a paucity of research exploring how relationships with household pets may impact maternal mental health. We are unaware of any study to date that has examined associations between individuals’ relationships with their pets and psychological adjustment in the perinatal period. Using a biobehavioral lens, this paper provides a narrative overview of the literature on perinatal mental health and human–animal interaction (HAI). We focus on the role of social relationships, stress, and stress reduction in relation to perinatal mental health; the role of HAI in perceptions of social support, stressors, and stress reduction; and gaps in empirical knowledge concerning the role of HAI in perinatal mental health. Finally, we integrate contemporary biobehavioral models of perinatal mental health and HAI (i.e., Comprehensive Model of Mental Health during the Perinatal Period and the HAI–HPA Transactional Model) to propose a new conceptual framework that depicts ways in which HAI during the perinatal period may influence maternal and child health and wellbeing. To our knowledge, this is the first paper to consider the role of HAI in biobehavioral responses and mental health during the perinatal period. We conclude with recommendations for future research and improved perinatal care.

## 1. Introduction

Empirical studies on the role of human–animal interaction (HAI) in human health and development emerged in the 1980s; since that time, the field of HAI has garnered increased attention and immense growth [1]. HAI refers to reciprocal interactions between a person and a non-human animal [2,3,4]. Research in this area often focuses on the benefits of the human–animal bond, which is defined as the, “mutually beneficial and dynamic relationship between people and other animals that is influenced by behaviors that are essential to the health and wellbeing of both” [5] (p. 1675). HAI is also considered to be a relational theory that describes human–animal dynamics that satisfy needs in each for companionship, emotional support, nurturing, and love [6,7,8]. It is important to note, however, that a majority of HAI science has focused exclusively on benefits to humans with few studies looking at the benefits to non-human animals. In addition, the literature is dominated by studies on human interactions with dogs and cats.

A prominent area of empirical focus within this field has been the impact of HAI on child development, due in part to the role social support, in general, plays in the promotion of positive child outcomes. The mechanisms through which household companion animals influence children’s physical and socioemotional health has become an increasingly popular topic within social and behavioral research. Indeed, nearly a decade has passed since the National Institutes of Health (i.e., National Institute of Child Health and Human Development) identified the implications of HAI in childhood as an important line of inquiry for developmental scientists [2,9]. Several studies suggest that HAI can have a positive impact on individual and social factors that influence child development. In particular, there is evidence that pet ownership and bonds with pets are associated with better emotional regulation and improved executive functioning, positive self-image, self-esteem, self-efficacy, and lower rates of loneliness in childhood and/or adolescence (e.g., [10,11,12,13]; see, for reviews [3,4]).

Some research, on the other hand, indicates that there are no differences between children who grow up with pets, and those who do not, and/or no association between HAI and measures of child development and adjustment (e.g., [14,15]). In addition, there is some evidence that aspects of HAI (e.g., bonds, attachment) are associated with increased feelings of loneliness, lower levels of human social support [16,17], and greater symptoms of psychopathology among youth [18] and emerging adults [19,20]. Despite inconsistencies across studies, there is increasing evidence that assessing children’s experiences with companion animals has important implications for understanding risk and resilience in child health and development [4]. What is not clear, however, is whether and to what extent HAI may indirectly influence child health and development via influences on caregivers’ health and wellbeing.

Interactions with companion animals can provide social support, companionship, and stress-buffering effects for adults; these effects have been documented in parenting samples [21,22,23,24,25,26]. However, living with companion animals is also associated with a host of unique stressors, and the risks and benefits of HAI may vary as a function of an individual’s social context and resources [27,28,29,30,31]. The broader literature on child development has long recognized the importance of caregiver mental health, especially maternal mental health during the perinatal period (i.e., the period spanning from the start of pregnancy to the first year following childbirth), in promoting the healthy development of children [32,33,34,35,36]. Yet, despite widespread evidence of links between perinatal mental health and children’s cognitive, behavioral, and psychomotor development, we are unaware of any studies examining how interactions with household companion animals may pose benefits and risks to maternal mental health during the perinatal period.

Perinatal mood and anxiety disorders (PMADs) are common complications of pregnancy [37]. Moreover, PMADs are a notable public health concern due to their deleterious short- and long-term impacts on maternal, child, and family outcomes [35,37,38]. It is estimated that the cost of not treating perinatal mental health problems among U.S. women exceeds USD 14 billion for all births when following the mother–child dyad for five years after birth [39]. Thus, understanding the potential effects of HAI on perinatal mental health has important implications for understanding how pets impact human health and development across generations.

### Overview of Current Paper

Using a biobehavioral lens, this paper provides a narrative overview of the literature on perinatal mental health and HAI with the goal of setting a research agenda that will expand knowledge of the role of HAI in human health and development during the perinatal period. We focus on the role of social relationships, stress, and stress reduction in relation to maternal mental health during the perinatal period; the role of HAI in perceptions of social support, stressors, and stress reduction; and gaps in empirical knowledge concerning the role of HAI in perinatal mental health. Finally, we propose and outline a new conceptual framework that visually depicts ways in which HAI during the perinatal period may influence maternal and child health and wellbeing. We acknowledge that the biobehavioral-HAI linkages reviewed in this paper may also be implicated in physical health outcomes; however, given the high prevalence of PMADs, we consider the role of HAI on biobehavioral responses specific to mental health to inform future research and practice within the context of the perinatal period.

## 2. Perinatal Mental Health

We begin by emphasizing that pregnancy experiences occur among many gender identities and expressions. In this paper, we use the terms “maternal” and “women” for simplicity and readability, and with consideration of the fact that a majority of studies in this area have focused on the perinatal experiences of cisgender women. PMADs have gained increasing recognition among clinicians and researchers due to their prevalence and significant morbidity during the perinatal period [40,41]. It is estimated that PMADs affect close to 20% of pregnant and postpartum women in the U.S., with rates increasing over the past decade [42], and more recently due to the impacts of chronic stress and social isolation associated with the COVID-19 pandemic [43,44]. Nationally representative data suggest that this rise in PMAD prevalence is likely a combination of genuinely increasing rates as well as enhanced awareness and screening in clinical and research settings [42].

Multiple studies link stress and PMAD development [45,46]. Stress is defined as the state of psychological or physiological imbalance that arises from situational demands that exceed the coping abilities of an individual [47,48,49,50]; stressors refer to emotionally or mentally disruptive conditions that alter homeostasis and lead to the release of regulatory hormones to return the body to homeostasis [51,52]. Several contextual factors put women at risk for increased levels of pregnancy-related stress and concomitant impacts on mental health. For example, age, minority stress associated with belonging to a marginalized racial/ethnic minority group [53,54,55,56], relationship status, economic insecurity and related factors such as housing instability and unemployment [57,58,59], work responsibilities, other caregiving responsibilities (e.g., children), and whether the pregnancy is planned or not, are well-documented factors that contribute to stress surrounding this major life event [60]. Other factors that relate to perinatal stress include, but are not limited to, social isolation and/or poor social support [61,62,63] and inter-partner conflict and violence [64,65,66].

Women with a life history of mental health challenges and prior exposure to adverse life events are at particular risk for poor psychological health during pregnancy [67,68,69]. Moreover, the intersectionality of social, economic, and educational disadvantage can be a critical risk factor for PMADs [70,71,72]. Women who are low-income are less likely to receive formal mental health treatment [73] and studies suggest that racialized women of color may be hesitant to seek treatment due to concerns regarding the stigma associated with mental health conditions [74,75]. In addition, recent findings suggest that there are short- and long-term health outcomes for the child who experiences the combined effects of the hardship of disadvantage and exposure to maternal depression [36].

### The Role of Psychoneuroimmunological Biomarkers in Maternal/Child Health

It has been recommended that researchers include psychoneuroimmunological (PNI) biomarkers (e.g., salivary cortisol levels, pro-inflammatory cytokines, oxytocin) to objectively assess and identify factors that increase or buffer risk of poor maternal/child health and to identify the mechanisms through which disadvantage, and the associated stressors, impact perinatal health [76]. The hypothalamic–pituitary–adrenal (HPA) axis, which includes the hypothalamus, pituitary gland, and adrenal glands, plays a key role in the biological response to stress [38]. In response to environmental (e.g., exposure to violence, poverty) and psychological (e.g., worry, anxiety) stressors, the HPA axis is responsible for activating the release of cortisol into the bloodstream which, in turn, signals for the release of necessary resources (e.g., glucose) to produce the “fight, flight, or freeze’’ response [38,77,78]. This process is generally adaptive and beneficial in response to typical acute exposures to stressors; however, prolonged exposure to stressors can result in dysregulated HPA axis activity, including both increased and/or blunted cortisol production. In some, but not all individuals, chronic stress or intense trauma may result in reductions in basal cortisol, alterations in typical diurnal cortisol patterns, and diminished or higher cortisol reactivity, which are consistent with exaggerated or unresponsive stress system responses and may manifest behaviorally in the form of shutting down (e.g., dissociation) [79,80,81,82]. Notably, for women in the perinatal period, these responses are associated with increased risk for more severe PMAD symptoms. For example, high cortisol in the early postpartum period has been linked with transient negative mood states (e.g., “baby blues”), whereas sustained low cortisol levels have been linked with chronic postpartum depression [83].

In addition to alterations in cortisol levels, exposure to chronic and/or overwhelming acute stressors have been associated with the dysregulation of oxytocin during the perinatal period [83,84,85]. Specifically, psychological stress has been implicated in disruptions in oxytocin pre- and post-partum [86], which has implications for maternal/infant attachment bonds [87,88,89] and breastfeeding success [84,90]. Indeed, disruptions to oxytocin during the transition to parenthood as a result of difficulties bonding or breastfeeding can compromise PNI functioning and increase risk of PMAD symptoms. For example, Cox et al. [84] found that, among breastfeeding women, those who reported clinically significant postpartum depression symptoms had lower oxytocin levels and higher cortisol levels during breastfeeding in comparison to asymptomatic women. Furthermore, dysregulation of the HPA axis response following a stress-induction task was positively associated with oxytocin levels among symptomatic women.

Research also links physical and psychological stressors in the perinatal period with inflammatory markers, which manifest via increased levels of pro-inflammatory cytokines. In fact, extant research documenting the bidirectional relationship between stress and immune system responses suggests that stressors (e.g., adverse childhood experiences) increase pro-inflammatory cytokines, which may, subsequently, contribute to mental health risk [80,85,91]. Indeed, it is argued that inflammation explains why various psychosocial and physical risk factors increase the risk for depression during the perinatal period. Specifically, both risk for depression and pro-inflammatory cytokines increase significantly during the last trimester of pregnancy and, therefore, women are particularly susceptible to the impacts of pro-inflammatory cytokines in the perinatal period. Studies examining psychological factors that contribute to cytokines in the perinatal period are lacking [92]. However, growing evidence indicates that pro-inflammatory cytokines are linked prenatally to anxiety and depressive symptoms [93,94], with a noted increase in the third trimester related to innate immune responses, as well as with changes in pro-inflammatory cytokines over the duration of the perinatal period [95].

#### Mother–Child Dyad

In addition to the direct effects on mothers, it is also important to consider the potential far-reaching effects of maternal stress and PMAD symptoms for the developing child and the mother–child relationship. The prenatal and early postnatal periods are recognized to be critical points in child development, related to stress physiology and long-term health and wellbeing [96]. Some research shows that intrauterine environments (i.e., fetal programming) characterized by high stress have consequences on children partly due to neuroregulatory and inflammatory mechanisms [97,98]. Due to the dyadic nature of the perinatal period, the intrauterine environment may be experienced as the very first early life stressor of the developing neonate. For example, maternal depression impacts the fetal environment in ways that may contribute to adverse birth outcomes (e.g., low birth weight), altered development and executive functioning [99], and poor physical and mental health across the lifespan [100]. Studies suggest that children of mothers with depression during the pregnancy have higher circulating cortisol [101] with associated alterations in the oxytocin receptor (*OXTR*) function [102,103]. Given what is known about maternal depression and *OXTR* function with regard to mother–child attachment and the ability of the child to adapt to psychologically stressful situations [104,105,106], these findings warrant close attention to interventions that can minimize maternal stress and depression during pregnancy and, thus, enhance outcomes for the child.

## 3. Social Relationships, the Stress Response, and Perinatal Mental Health

One method of facilitating an adaptive stress response is to modify how stressors are perceived [38]. For example, decreasing the level of perceived stress can prevent activation of the HPA axis altogether, whereas increasing the level of perceived ability to cope with stress can aid in regulating the stress-response following HPA axis activation [107,108]. The level of perceived socio-emotional support influences these processes, such that higher levels of perceived support aid in buffering an individual’s response to stress [109,110,111]. It is well established that a sense of social connectedness and social group membership can be highly protective against perinatal depression, stress, and other psychological symptoms [63,112,113]. For example, in a 2017 study of close to 400 perinatal women in the U.S., new mothers who had a higher number of social connections during the perinatal period experienced lower rates of depressive symptoms [114]. Importantly, a lack of perceived social support and social connectedness can not only contribute to the development of depressive symptoms but can also be an outcome of such symptoms, essentially trapping individuals in a vicious cycle [115].

There is no “one-size-fits-all” coping strategy that is known to work for all individuals experiencing stress; however, research efforts are needed that focus on developing interventions aimed to enhance a sense of coping in the face of stressful situations and thus decrease risk of the development of PMADs. Relationships with household pets may serve as a possible support to help alter perceptions of stress and assist with healthy psychological and physiological coping under conditions of stress during the perinatal period. Given the extant literature on the protective role of social relationships with other humans, in the next section we consider how social relationships with non-human animals, specifically household companion animals (e.g., pet dogs, cats), may serve as a protective social relationship during the perinatal period. We also identify the mechanisms through which HAI may influence perinatal mental health via stress reduction.

### Potential Benefits of HAI during the Perinatal Period

Similar to the effects of social relationships with other humans, people may experience an enhanced sense of emotional safety in the presence of their companion animals [116]. Approximately 60% of U.S. households report having at least one pet, with dogs and cats being the most prevalent [117]. A majority of people who live with pets consider them to be important social relationships and a member of their family [118]. Indeed, many individuals perceive that companion animals are more reliable sources of socioemotional support than humans; this is particularly true among marginalized populations impacted by adverse social relationships and environments [119,120,121,122]. To this end, there is some evidence that pet ownership and other aspects of HAI (attachment to pets, positive engagement with pets, emotional comfort derived from pets) may help to mitigate the deleterious impacts of adverse experiences and stress on psychological wellbeing (i.e., anxiety and mood disorder symptoms; [29,120,123,124,125]). For example, prior studies provide evidence that: HAI may function as a protective factor that buffers the relation between intimate partner violence exposure and internalizing symptoms in children [123]; emerging adults seek out HAI as a coping strategy following exposure to sexual and gender minority stressors and, in turn, HAI fosters personal hardiness following adversity [126]; HAI buffers the impact of victimization on self-esteem among LGBTQ+ emerging adults [29]; and adults who experience familial abuse and live with an animal report less psychological distress than adults experiencing abuse who do not live with pets [124]. Despite these advancements in the literature regarding the benefits of HAI in relation to mental health, the biobehavioral mechanisms that explain these relations have yet to be rigorously explored.

The potentially protective aspects of HAI may stem, in part, from their impact on an individual’s perceived social support. There is some evidence that pets can facilitate interactions with other humans [120,121,127,128], which may lead individuals to perceive a greater sense of overall social support, inclusive of human and non-human animals. Relationships and bonds with companion animals may also help to ameliorate loneliness and remedy the negative impacts of social isolation [129,130,131,132,133]. It is also important to highlight that pets provide humans with a sense of belonging, which is hypothesized to be associated with increased perceptions of social support [120,134,135,136,137]. These benefits may be further enhanced through physical contact with animals, which is often viewed as a behavioral expression of attachment bonds and social support [138,139].

Although the collective body of research has produced mixed evidence for the benefits of HAI, it is not surprising, given the potential benefits outlined above, that some studies link pet ownership and aspects of HAI (e.g., attachment, bonds, caretaking) with stress reduction [140,141,142,143], greater physical activity and overall better physical health [144,145,146,147,148,149], and higher levels of self-esteem and self-efficacy [148,150,151] at multiple stages of human development. In addition, there is some evidence that short-term HAI via animal-assisted activities and interventions may reduce the risk of, and symptoms of, specific adult mental health problems, such as anxiety [152,153] and depression [154,155]. For example, a 2007 meta-analysis by Souter and Miller [156] identified five studies that utilized animal-assisted interventions to treat depression. The results suggest that HAI is associated with decreases in depressive symptoms. Additional studies with older adults living within care facilities provide evidence that caring for an animal (in these studies, a bird) is associated with less depressive symptoms [157,158]. Similarly, Barker et al. [159] found that, among patients waiting for a psychiatric procedure, those who interacted with an animal reported significantly lower levels of fear and anxiety in comparison to patients within a control group.

Although some studies have focused on the implications of HAI for child development, minimal attention has been given to how pets may indirectly influence child health and wellbeing via influences on adult caregivers’ health and wellbeing. Moreover, few studies have examined the direct effects of HAI on mental health and related outcomes in parenting samples. However, in two intervention studies involving parents of children with autism spectrum disorder who acquired a pet dog as an intervention, parents reported significantly less stress than the control group without a pet dog [23,26]. We are aware of only one study that has examined interactions with animals among women during the perinatal period. Specifically, Lynch et al. [153] conducted a pilot study to examine the use of “pet therapy” (non-structured in-room contact with a dog) among women who were hospitalized due to high-risk pregnancies (e.g., hyperemesis, preeclampsia) and found that self-reported anxiety and depression symptoms decreased following a session with a therapy animal. Given the importance of supportive caregiving environments in the promotion of positive child development [160,161,162], improving maternal mental health via HAI may be an essential pathway to enhance maternal sensitivity and responsivity, thereby affecting later mother–child relationships and children’s developmental trajectories.

Collectively, the literature suggests that both short-term (e.g., animal-assisted interventions) and long-term (e.g., relationships and interactions with household companion animals) HAI warrant examination as a means of promoting psychological health in the perinatal period. However, the mechanisms that underlie the relationship between HAI and mental health during the perinatal period are unknown, and such an understanding would provide key targets for early intervention to promote positive outcomes across two generations—mothers and their children. In response to calls to evaluate whether/how to integrate biomarker measurements into research on family mental health (e.g., [76]), attention must be paid to biomarker work in the HAI field. Contemporary biobehavioral perspectives of HAI and related studies are reviewed below.

#### Biobehavioral Underpinnings of HAI

Similar to the field of perinatology, there have been calls and efforts to integrate biomarker measurement into research on HAI (e.g., [139,163,164,165]). However, the integration of biobehavioral measures in HAI science remains an underdeveloped and emerging area of the field. There is growing evidence that human–animal bonds involve important biological pathways that play critical roles in mammalian social behavior and emotion [27]. Specifically, it is hypothesized that there are alterations in the autonomic nervous and neuroendocrine systems from HAI and that oxytocin and vasopressin act as neurotransmitters and neuromodulators that underlie bonds between humans and their pets [27,116,166,167]. Indeed, research suggests many potential mechanisms through which HAI impacts humans’ emotional and physiological state wellbeing, including touching (petting), gazing at, and affiliative contact with pets [138,139,140,143,168,169]. For example, studies of adult samples link petting animals with lower heart rate and/or blood pressure [170,171,172,173]; increased immunoglobulin A, β-phenylethylamine, oxytocin, and dopamine [173,174,175]; and lower levels of cortisol (lower cortisol stress response; [173,175,176,177]).

Despite limited research testing hypotheses regarding the biobehavioral processes through which HAI is beneficial to human health and wellbeing, a few studies on short-term HAI indicate that HAI can produce changes in biological stress systems. It is largely assumed that HAI buffers stress via decreased cortisol both prior to and after activation of the stress response system, and a key component of this effect includes perceived social support provided by pets [139]. For example, a recent study found that among children who completed the Trier Social Stress Test (a laboratory-based stress induction task), those who were randomly assigned to complete the test with their dog present had lower cortisol levels following the stress test than children who had their parent present and children who had no support during the test [178]. Polheber and Matchock [179] found similar results among a sample of adults who completed the Trier Social Stress Test; adults who completed the test with a novel dog had lower cortisol levels in comparison to those who had a human friend present during the task and those who had no support during the task. These findings suggest that animals may serve as important protective factors for individuals under conditions of stress [142,143,167,173].

Prior research also links gazing at and/or petting animals with increased oxytocin and/or vasopressin concentrations in adults [143,169]. Beetz et al. [140] proposed that the release of oxytocin, as a result of close/affiliative bonds with a pet, may mediate relations between HAI and positive outcomes, such as increased social interactions [17,180,181], decreases in cortisol levels [182], and improved mental health (e.g., decreases in anxiety and depressive symptoms; [183], see for a review [156]). In addition, stress-response benefits associated with oxytocin release are hypothesized to occur as a result of HAI due to the presence of a pet decreasing perceived threat [82,184,185]. Threat appraisal stimulates the stress-response system; as previously mentioned, in the absence of adequate social support, the nervous system resorts to “fight-or-flight”, and potentially “freeze” or immobilization, which can lead to dissociation [82]. The presence of, and interaction with, a companion animal may release oxytocin (see for reviews [139,140]) and provide humans with the necessary social support and comfort to create a sense of safety and regulation [186,187], which aids in disrupting the stress-response system.

It is important to note that these benefits may also depend on variation in genetic predisposition to oxytocin sensitivity. Although there has been limited empirical research in this area, there is some evidence to suggest that *OXTR* genotype (possibly affecting the expression of the oxytocin receptor and thus sensitivity to oxytocin) is linked with natural variation in interactions with dogs. For example, Kertes et al. [188] examined whether the *OXTR* genotype was related to children’s perceived relationships and their petting and gazing behaviors with pet dogs. Simulating naturally occurring HAI in the context of a laboratory experiment, the results of this study indicated that variation at the *OXTR* polymorphism rs53576 was associated with the proportion of time spent petting during child–pet interactions, but not gazing behaviors. On average, all children in the sample spent 50% of a 10 minute interaction petting their pet; however, A-carriers engaged in significantly more petting behavior than children with the GG genotype. This is an important finding, given that previous research suggests that A-carriers (individuals with the AA or AG genotype) are less responsive to parental support and peer relations than those with the GG genotype (e.g., [189,190]); A-carriers also demonstrate lower levels of interpersonal empathy, trust, and self-esteem [191,192,193]. Such findings complement other research documenting that children with anxiety disorders tend to interact with a pet dog for long durations and have fewer interactions with other people compared to children with other behavioral health problems [194]. Although there is significant need for replication of the Kertes et al. [188] study in child and adult samples, this suggests that pets (i.e., dogs) may be important sources of social interaction for individuals with socioemotional difficulties or challenges connecting with other humans. Moreover, *OXTR* genotype may be a biomarker that warrants attention in studies examining the role of HAI in perinatal health.

For individuals who have experienced trauma, high levels of stressors, and/or experience difficulty engaging with other humans (e.g., those with social anxiety disorders), interactions with companion animals may provide critical social support [120,187,195,196]. Indeed, it is hypothesized that the benefits of HAI may be most pronounced when individuals face adversity and chronic stress, referred to as a “stress state” ([116] (p. 98), see also [31,197]). Companion animals are often reported to be nonjudgmental, unconditional sources of support [116,198], which may amplify the utility of the socioemotional support they provide to humans. Although there are studies that have examined the effects of pets on the stress-response (e.g., cortisol, oxytocin) within people’s home environments (e.g., [142]) and/or over time (e.g., [199,200]), the majority of HAI biobehavioral research, as evidenced in the current review, is limited to single sessions (e.g., [176,177]) and controlled environments (e.g., laboratory setting or college animal-visitation program; [178,179,188]). Given the stress-buffering benefits following short-term exposure to companion animals (via animal-assisted interventions), it is possible that repeated exposures over time, through everyday interactions with pets at home, may promote the down-regulation of the stress response system (i.e., HPA axis), a return to homeostasis, and ultimately improve mental health during the perinatal period [139].

It is also important to consider that animals that are typically involved in short term interventions have often met specific training requirements and do not exhibit behavioral issues and challenges that are common among many household pets. Thus, many theoretical models that aim to explain the benefits of HAI (beyond animal-assisted interventions) fail to recognize the unique characteristics of HAI and pet ownership that may contribute to and/or exacerbate stress and create barriers to wellbeing. We caution against the assumption that pets are equivalent to therapy animals or that pets should be used or acquired as an intervention to prevent and/or treat mental health symptoms. Recent studies suggest that pets may in fact contribute to an individual’s stress state and/or exacerbate existing stressors [19,120,201]. In order to better assess the long-term biobehavioral benefits of HAI on mental health outcomes, HAI science should consider a more nuanced perspective of the role of interactions with household pets, inclusive of the role of pets in acute and chronic stress and trauma [27]. In the next section, we outline the ways in which HAI may be associated with risk for PMADs during the perinatal period.

## 4. HAI and Potential Risks to Perinatal Mental Health

As previously addressed, experiencing stress during pregnancy is common and often compounded by typical mobilizing emotions such as excitement and fear. To our knowledge, there is currently no literature concerning the potential negative impacts of pet ownership and related aspects of HAI on perinatal mental health. Given the dearth of research on this topic, our discussion focuses on how known risks of HAI (i.e., companion animals/family pets) may negatively impact mental health by causing and/or compounding stress via caregiving burden, problems related to pets (e.g., behavioral problems), and social/environmental factors that disadvantage certain populations of pet owners and guardians of human children alike.

There are several factors related to pet caregiving that could, hypothetically, contribute to or exacerbate stress and, therefore, be detrimental to mental health during the perinatal period. In a qualitative study, adult pet owners reported negative emotions (i.e., stress, sadness) associated with their pets’ behavioral problems, such as separation anxiety, inappropriate elimination, and aggression [202]. Kertes et al. [188] found that children who reported greater frequency of negative interactions (i.e., annoyance) with their pets spent less time petting and engaging with their pet dog compared to those who reported less negative interactions. The results of these studies highlight the potential for negative aspects of pet ownership to jeopardize the human–animal bond, decrease time pet owners spend interacting with their pet (which has previously been discussed as a potential mechanism of stress reduction), and significantly impact the mental health and wellbeing of the owner. Especially in the case of a first child to new parents, behavioral issues in pets may cause feelings of anxiety and guilt if the behavioral concerns could be related to the new baby’s health and safety. For example, first-time parents may fear that their pet could become unpredictable or aggressive toward an infant, particularly if the pet has exhibited fear or reactivity in the past; the sights and sounds of a new baby can be disturbing to a dog or cat, especially if novel [203]. Further, though the literature is unclear concerning the actual risk [204,205], the possibility that a new baby could exhibit asthma or atopic symptoms in response to pet dander is something that a new parent might have to grapple with. During the prenatal period it is typically advised that the pregnant person limit interactions with cats, including those that live in their household, due to the risk of toxoplasma infection and subsequent risk of harm to the fetus [206,207]. For an owner who derives emotional comfort from their cat, limiting interaction due to toxoplasmosis risk could cause considerable distress. Practitioners should also consider the impact the loss of a pet (rehoming and/or death) during the perinatal period may have on mental health. There is evidence that caring for a terminally ill pet is associated with increased stress and anxiety and depressive symptoms and lower quality of life [208,209,210] and subsequently the death of a pet is linked to anxiety, depression, and overall psychological distress [211,212,213].

For low-resourced individuals, the costs associated with both the perinatal period and pet caregiving can cause considerable strain within household budgeting. Indeed, veterinary care and pet supplies are expensive [214], and considerable resources (both social and economic) are necessary for caring for a pet. Perhaps most illustrative of this is the inaccessibility of pet-friendly rental housing, which is known to be both more expensive and less prevalent than pet-restrictive housing, particularly in racialized communities of color [215,216]. Residential mobility is common during the perinatal period, particularly for low-income and racialized minority individuals [217]. The combined stressors of finding an affordable new home that is both appropriate for a new baby and will also allow one’s pet(s) could prove substantial.

Beyond economic resources, social resources are also an important factor in both baby and pet caregiving. For example, recent research regarding pet caregiving during the COVID-19 pandemic revealed a strain on household resources (both social and economic) in order to plan for the potential of caregiver hospitalization or incapacitation [218]. In particular, pet owners with children identified their social networks as contingency care plans in the event of caregiver illness [219]. Even during typical (i.e., pre/post pandemic) times, balancing caregiving roles between pets and a new baby can be a challenge for those who lack the resources to rely on help from paid caregivers and/or their social network. Social and economic resources may also provide the ability to rely upon support from one’s social network, or paid care (such as a postpartum doula or nanny), to support adequate sleep for new parents despite the overnight caregiving demands of a newborn. Indeed, sleep disturbances in the perinatal period are known contributors to poorer mental health [220,221]. Similarly, pets may contribute to sleep disturbances, which could, in turn, exacerbate perinatal sleep problems. Although the literature is conflicting, research seems to suggest that pets are likely to disturb sleep, but may provide a sense of safety and security, which, in turn, may improve sleep [222]. Furthermore, the transition to parenting may increase stressors that result in conflict between caregivers and exacerbate the risk of intimate partner violence (IPV; [223]). It is well documented that IPV and animal cruelty commonly co-occur [224,225,226,227]. As such, pets of adult and child IPV survivors may be a target for animal cruelty, which may, in turn, lead to negative impacts on caregiver and child outcomes. Collectively, this research highlights important contextual factors to be considered in the role of HAI during the perinatal period. Although outside the scope of the current paper, it is important that future studies consider how these perinatal stressors and related behaviors can have detrimental effects on the welfare of animals.

## 5. Discussion

We end with a discussion of opportunities for integrating two recently proposed conceptual models—one from the field of perinatology and the other from the field of HAI—to guide research at the intersection of perinatal mental health, child development, and HAI. Specifically, we review Moyer and Kinser’s [228] Comprehensive Model of Mental Health during the Perinatal Period and Pendry and Vandagriff’s [139] HAI–HPA Transactional Model. First, we summarize key concepts from the Moyer and Kinser model and HAI–HPA Transactional model. We then expand on these key concepts to create an integrated model that outlines key research questions and hypotheses to guide future research on the role of HAI in maternal and child health and development with attention to multispecies family-centered care.

### 5.1. Key Concepts from the Comprehensive Model of Mental Health during the Perinatal Period

The Comprehensive Model of Mental Health during the Perinatal Period [228] is helpful in guiding research at the intersections of perinatal mental health, HAI, and child development. This model addresses the interplay of various biopsychosocial and PNI pathways linking individual and dyadic maternal and child health. The three main principles of the Moyer and Kinser model highlight the importance of: (1) the biopsychosocial and PNI mechanistic pathways involved in the development of perinatal mental health (reviewed above in Section 2), (2) the role of individuals’ experiences of matrescence in perinatal mental health, and (3) the importance of considering the mother–child dyad as a functional unit [228]. For example, matrescence experiences vary greatly and can contribute to additional stress during the perinatal period [229]. We aim to advance Moyer and Kinser’s [228] model by proposing future research that focuses on understanding how pet ownership and related aspects of HAI influence stress reactivity, inflammation, and HPA axis activity in the perinatal period (e.g., perceived reductions or increases in stress due to household pets) and in relation to all members of the family system.

### 5.2. Key Concepts from the HAI–HPA Transactional Model

Building on prior HAI research and knowledge of the biological stress response system, Pendry and Vandagriff [139] proposed the HAI–HPA Transactional Model, a framework that details how HAI attenuates the stress response system, ultimately disrupting the association between stress and mental health problems (e.g., anxiety/depression) via PNI mechanisms. According to their model, which centers on the role of touch and socioemotional support provided by companion animals in short-term animal-assisted interventions, HAI buffers stress both prior to and after activation of the stress response system. As discussed previously, the socioemotional support provided by pets may aid pet owners in perceiving potential stressors as less threatening, which may prevent the activation of the stress response system completely. However, not every stressor will be attenuated by interactions with companion animals. Once the stress response system is activated, pets can continue to boost perceptions of support, allowing humans to re-appraise whether their situation is still stressful. Moreover, people may facilitate interactions with their pets in a way that serves as a catalyst for ameliorative effects (e.g., increases in oxytocin and decreases in cortisol) via the downregulation of physiological arousal and cortisol production via the HPA feedback loop. Although this model focuses on what Pendry and Vandagriff consider to be the main marker of HPA activity, cortisol, they also address links between HAI and alpha amylase, secretory immunoglobulin A, oxytocin, testosterone, and nerve growth factor [139].

The HAI–HPA Transactional Model provides a useful framework for how HAI may influence perinatal mental health outcomes via the stress-response system. However, it is centered on evidence and characteristics of short-term animal-assisted interventions for individuals rather than the unique characteristics of HAI involving household pets within the family system. Moreover, this model does not adequately attend to the role of social context in shaping relationships with animals and responses to HAI. We are unaware of any contemporary model that accounts for the biobehavioral benefits *and* risks of pet ownership and related HAI with the goal of identifying the mechanisms through which HAI shapes mental health.

### 5.3. Integrating Models to Guide Research on the Intersection of HAI and Perinatal Health

**Figure 1:**
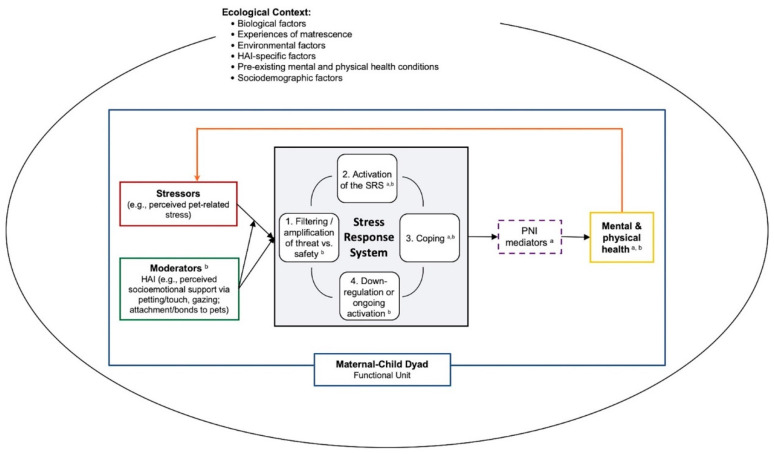
Human–animal focused integrative model of stress and perinatal mental health. Dashed lines reflect that PNI mediators may not always be present in the model. For space and clarity, we did not specify individual ecological factors in the figure. Some examples of biological factors may include epigenetic patterns, evolutionary history, endocrinology of selective social bonds; environmental factors may include experiences of adversity and/or discrimination (e.g., adverse childhood experiences, intimate partner violence, racism); HAI-specific factors may include location (home, community, laboratory), dosage (frequency, duration, intensity), species type, method (HAI involving household pets, HAI through animal-assisted interventions); and sociodemographic factors may include age, sexual orientation, gender identity, race/ethnicity, geographic location. HAI = human–animal interaction. SRS = stress response system. PNI = psycho–neuro–immunological. ^a^ Moyer and Kinser’s Comprehensive Model for Mental Health during the Perinatal Period. ^b^ Pendry and Vandagriff’s HAI-HPA Transactional Model.

Building on prior HAI and perinatal research, it is important to examine the role of interactions between people and their pets in the hypothesized biobehavioral pathways elucidated in the Moyer and Kinser model and to tease apart the effects of support from pets and human forms of social support (which can also be shaped by HAI). In the following sections, we integrate key elements from the HAI–HPA Transactional Model [139] and Moyer and Kinser [228] model into a single conceptual model (see Figure 1), and discuss how household pets may influence the stress response system (i.e., buffering and/or exacerbating stress), the transition to motherhood, the development of the infant via maternal mental health, and the mental health of other caregivers. Finally, we consider how perinatal mental health and the role of HAI during this period may vary based on the ecological context. A visual representation of this integrated model is shown in Figure 1. In brief, our “Human-Animal Focused Integrative Model of Stress and Perinatal Mental Health” provides a framework for examining the role that companion animals may play in the perinatal period; specifically, it emphasizes the role of HAI in buffering and/or exacerbating stress and the stress response system, and in turn, impacting PMAD symptoms via PNI mediators. We highlight the importance of assessing two key aspects of HAI—perceived socioemotional support (including behavioral expression such as touch and gazing) and perceived pet-related stress—within a comprehensive model of perinatal health. This model also emphasizes the importance of examining these processes within the ecological contexts of the family unit.

#### 5.3.1. HAI and the Stress Response System

Future research on the role of pets in perinatal mental health should examine how pets, as part of the family system, influence coping with stress. Studies are needed to determine the role of physical touch and socioemotional support from pets in the stress response system of mothers during the perinatal period. Interactions with pets (e.g., petting) may serve as a coping mechanism by providing a distracting activity while also providing biobehavioral benefits [230,231]. To this end, studies are needed to identify the PNI processes/mechanisms through which emotional support gained from interactions with pets may attenuate the stress-response system. Specifically, longitudinal studies can aid in elucidating what aspects of HAI (e.g., touch, socioemotional support) may lead to better mental health outcomes, and whether those benefits are in fact due to changes in the stress response system (e.g., lower cortisol levels, higher oxytocin levels) and/or the inflammatory processes [232]. It is also necessary to consider the reciprocal relationship between HAI and biobehavioral benefits, as higher oxytocin levels may promote the establishment of close bonds with pets which, in turn, may result in more frequent HAI. Additionally, researchers should consider how HAI is measured (e.g., frequency of interactions, perceived level of benefit) in these studies as different conceptualizations of HAI may be differentially associated with perinatal mental health outcomes.

As previously noted, many of the hypotheses regarding the role of HAI are centered on benefits to mental health, perhaps due to the focus on short-term (i.e., animal-assisted interventions) interactions with animals instead of long-term (i.e., pet ownership) and intimate interactions with animals. As previously mentioned, this is a limitation of the HAI–HPA Transactional Model [139], which does not consider how the presence of an animal, particularly one to which a person is bonded, may increase stress and/or threaten appraisals (while simultaneously having the capacity to attenuate the stress response system). Therefore, we base our recommendations on future research regarding how pet ownership and HAI may negatively impact perinatal mental health on the existing evidence reviewed previously. Research is needed to identify how pets may contribute to the activation of the stress-response system during the perinatal period and how to eliminate these pet-related stressors. Additionally, longitudinal research that examines how pet-related stress may change over time and when it may have greater impact on perinatal mental health is needed to determine if there may be critical periods when expectant parents may need additional support or may be most at risk for PMAD symptoms. Researchers and practitioners may consider how assisting a family in finding reliable pet care or behavioral services in advance of the (often unpredictable and sometimes emergent) birthing process may help relieve some pet-related stressors. Research should also consider the changing dynamics of relationships with pets when a new baby is introduced into the household. For example, a new baby may cause strain within the family, which potentially jeopardizes the pet’s welfare and possibly even threatens the pet’s role within the family unit. Further, if HPA activation is dependent on the characteristics, severity, and frequency of daily stressors, and also on appraisal processes, it would behoove future researchers examining the role of HAI in perinatal mental health to explore the characteristics of pet-related stressors, appraisals of those stressors, and how aspects of HAI may impact the appraisal of co-occurring stressors [139,233].

Moreover, most hypotheses regarding the benefits of HAI fail to consider how the benefits and risks associated with companion animals may vary as a function of an individual’s social resources. Therefore, examining how pets may increase or decrease human social resources is important within the context of perinatal mental health. For example, pets can be a barrier to social relationships due to pets’ behavioral problems (e.g., aggressive behavior) and other people’s allergies [120]. Given the benefits of perceived and received human social support for parental mental health and child health [114,234], it is important for practitioners and researchers to better understand how pets may influence available support during the perinatal period and what resources are needed if human social support is lacking during this period.

Extant research is also limited with respect to biobehavioral assessments and methodology within the context of HAI and health outcomes. For example, evidence suggests changes in the hormone cortisol in the presence of a dog during laboratory settings [178,179]. More studies are needed to assess other indices of stress, such as heart rate variability or oxytocin, to better understand the mechanisms underlying the relationship between HAI and mental health during the perinatal period. Importantly, however, ecological valid assessments are also warranted to determine the role of animals on multiple biomarkers of stress [197]. Research that examines alterations in the stress response system in naturalistic settings, such as in families’ homes, may help to identify momentary and real-world experiences with pets that can ultimately shape maternal and child health and wellbeing.

#### 5.3.2. HAI and Matrescence

The Moyer–Kinser model includes the process of matrescence as a key factor that may influence maternal mental health and child development through the stress associated with the transition to motherhood [228,235,236]. Prior pet ownership may aid in this transition, as expectant parent(s) may have previously had to adjust their routines and responsibilities upon obtaining a new pet, which may help them to navigate the demands (e.g., responsibility for the infants’ needs, financial, psychological worry over infant’s wellbeing) and rewards (e.g., purpose, emotional fulfillment) associated with parenthood while balancing their own needs (e.g., sleep, return to work; [237,238,239]). Future research should consider how adjustment to pet ownership may predict adjustment to parenthood; in line with holistic, individualized care, practitioners can draw upon expectant parents’ pet owning experiences to help them prepare for and adjust to the new baby by identifying their available resources and potential needs.

Additionally, pets may further aid new parents during the perinatal period and in the postpartum transition through promoting associative bonding. As discussed previously, interactions with pets, especially those involving touch, may increase oxytocin levels in humans. Given the role of oxytocin in maternal-infant bonding and successful lactation postpartum [84,87,88,89,90] and the mixed results regarding the effectiveness of intranasal oxytocin in improving lactation [240,241], researchers should also consider how oxytocin increases as a result of HAI may influence maternal-infant bonding and lactation. Perhaps the natural release of oxytocin from interactions with pets may have a greater, more consistent effect than intranasally administered oxytocin; however, this has not been tested to our knowledge. For those who wish to breastfeed and cannot, this experience may be a source of stress, which HAI may buffer through the provision of emotional support. Taken together, HAI may provide insight into the variation in experiences of matrescence and highlights how considering pets as part of the functional family unit may help to prepare and support expectant mothers during the transition to motherhood.

#### 5.3.3. HAI and the Mother–Child Dyad

Investigating the role of pets and HAI in supporting maternal mental health during the perinatal period has indirect implications for the developing child. Moyer and Kinser [228] emphasize this connection between maternal mental health and wellbeing and infant development and wellbeing, and argue that the mother–child dyad should be considered a functional unit in terms of perinatal care. Although there is existing research supporting the link between stress experienced by the mother (e.g., IPV, general parenting stress), maternal mental health problems, and developmental outcomes (e.g., internalizing and externalizing behaviors) in children across developmental periods [99,242,243,244], to our knowledge there are no HAI studies that have examined how HAI may influence these relations [4]. Longitudinal research is needed to better understand how HAI may indirectly affect infant development during the perinatal period.

First, researchers should examine how HAI may buffer and/or exacerbate prenatal mental health symptoms via the biological embedding of stress, and how these effects, in turn, may impact birth outcomes and infant development. This is critical as prenatal maternal stress (i.e., higher levels of cortisol and pro-inflammatory biomarkers, such as IL-6) in utero and postpartum have been associated with higher infant cortisol levels and stress reactivity [245,246] and neurocognitive developmental outcomes (e.g., structural brain alterations) that may increase risk for long-term behavioral problems [247,248]. Second, it is important to consider how HAI may improve parent–child interactions and child development. For example, if HAI buffers maternal stress, then mothers may be better able to focus on bonding with the infant and have more positive, engaging interactions with their infant. Positive affiliative bonding between mother and child is associated with oxytocin releases in the mother–child dyad, which is linked with secure attachment patterns for the infant. Moreover, children exhibit less cortisol reactivity to stressors when mothers employ more supportive and responsive caregiving, which in turn is associated with positive socio-emotional outcomes [161,249]. Emerging research also suggests that the coupling of mother–infant physiology and behavior (i.e., coregulation) varies by environmental factors [250,251]. For example, cortisol synchrony, or the degree to which mother–child cortisol levels are mutually regulated within a dyadic context, is shown to reduce the risk of children’s internalizing symptoms [252]. Therefore, if pets protect against mothers’ maladaptive responses to stress and PMAD symptoms, this may in turn impact maternal-infant biobehavioral coregulation and associated child outcomes. In future work, examination of the consistency of supportive and responsive caregiving in the presence of animals as related to infant physiology and developmental outcomes may be an important advancement in understanding the role of HAI in the context of mother–child dyads. This may be especially critical among families characterized by high-risk environments when there may be differences in positive parenting behaviors due to exposure to multiple stressors.

##### HAI and Other Caregivers

Although the Moyer–Kinser Model specifically discusses the “maternal-child dyad”, it is important to expand the focus to consider the entire family as a functional unit, such as partners or other secondary caregivers [253,254,255], and how HAI may impact other caregivers within the family unit. Secondary caregivers have been implicated in maternal mental health and in the infant’s long-term behavioral and emotional development [40,256,257,258]. Just as HAI may provide stress-buffering benefits to maternal mental health, HAI may also provide these same benefits for secondary caregivers in adjusting to additional stress during the perinatal period. For example, HAI studies examining the benefits of service dogs for military veterans diagnosed with post-traumatic stress disorder provide support that the benefits of animals in the home can extend to the family unit [259,260]. Partners of veterans described how the service animal provided a shared activity, socioemotional support, and supported their own quality of life and mental health [259,260]. Therefore, it is important to consider how HAI may influence family members outside of the mother-child dyad given that prior research documents relations between primary and secondary caregiver’s depressive severity across the first 6 months postpartum. Paulson et al. [257] found that mothers with partners who met depression criteria during the prenatal period were 4.2 times more likely to have higher depressive severity at 6 months compared to mothers whose partners did not meet depression criteria. However, mothers’ prenatal depression status did not significantly predict changes in their partners’ depression during the postpartum period [257]. This suggests that promoting secondary caregivers’ mental health during the perinatal period may, in turn, support maternal mental health. Further, focusing on supporting secondary caregivers’ mental health may also allow them to be more available to support the mother during this period, and greater support from a partner is significantly associated with lower rates of PMADs [261]. It is, therefore, important to consider the role of pets in secondary caregivers’ experiences of stress and support during the perinatal period, as well as how pets may influence the relationship between primary and secondary caregivers.

Moyer and Kinser’s [228] model can also be expanded to include alternative and less prevalent perinatal scenarios when one (or both) parts of the mother–child dyad are missing, such as surrogacy, parental/infant death, and child protective services involvement. Such scenarios highlight the importance of considering how to best holistically support all individuals involved in the caregiver-child dyad, even if those individuals are no longer part of a mother–child dyad. For example, in the case of a surrogate or a birthing parent whose child is placed into foster care following birth, the birthing parent still needs postnatal care regarding how to adapt postpartum without the child. Current research and medical practice focus on postpartum care for birthing parents actively caring for the infant; however, ensuring that birthing parents are supported in adapting and coping post-birth, especially considering the potential additional stress involved with being separated from a child, has implications for their mental health. Given the role of pets in coping with trauma and adversity, the role of HAI warrants attention as a factor that may facilitate coping in these alternative and less prevalent scenarios.

#### 5.3.4. HAI within the Ecological Context

Finally, we end by emphasizing a key concept of the Moyer and Kinser model [228] that is often neglected in the design and interpretation of HAI research: the ecological context. As emphasized in Moyer and Kinser’s Comprehensive Model of Mental Health during the Perinatal Period [228], there are a number of individualized biological and environmental factors that may increase or decrease risk for PMADs (e.g., epigenetic patterns, exposure to adversity and discrimination, PNI mediators). We specifically highlight the importance of including social context and adversity in future studies examining the role of HAI in perinatal mental health. For example, low-income families may be at risk for experiencing additional finance-related stress during the perinatal period as they prepare to care for the new baby. This stress experienced by low-income, pet-owning families during the perinatal period may contribute to disparities in maternal mental health outcomes based on socioeconomic status. Researchers examining pet ownership and HAI during the perinatal period should examine differences in the risks and benefits of HAI on perinatal mental health based on socioeconomic status and levels of financial stress [30]. In addition, future studies should consider how pet ownership and HAI may exacerbate and/or buffer risk for PMADs in the context of exposure to childhood and current adversity (e.g., domestic violence, adverse childhood experiences, racism), given that biobehavioral processes and the impact of HAI may differ in the context of chronic adversity [31,116,262]. It is critical that future researchers and practitioners intentionally and appropriately explore how pets and HAI factor into variations in ecological context to delineate how, for whom, and why HAI poses risks and benefits in the perinatal period.

## 6. Conclusions

This paper examines the potential role of HAI in ameliorating and exacerbating PMADs and potential indirect effects of HAI on the developing child and family unit. There is a substantial need for nonpharmacological and accessible therapies that engage individuals in self-management to address PMADs. As previously mentioned, in the U.S., the deleterious outcomes associated with not treating perinatal mental health problems is estimated to exceed USD 14 billion for all births when following the mother–child dyad for five years after birth [39]. Practitioners and researchers should explore how relationships and activities with household pets may be employed to support maternal mental health during the perinatal period, such as through companionship and stress reduction via biobehavioral processes (e.g., reduction in cortisol, release of oxytocin). Understanding the role of pets in perinatal stress and resilience will help the field of HAI advance toward better understanding the role of pets in child and human development. Further, the role of pet ownership and pet behavior in social and economic disadvantages during this time has implications for the wellbeing of the mother–child dyad and family unit. Thus, to address health disparities in mother–child health, it is critical to attend to these key issues regarding the role of pets in buffering and exacerbating the risk for and burden of PMADs.

## References

[1] Rodriguez K.E., Herzog H., Gee N.R. (2021). Variability in Human-Animal Interaction Research. Front. Vet. Sci..

[2] Esposito L., McCune S., Griffin J.A., Maholmes V. (2011). Directions in Human-Animal Interaction Research: Child Development, Health, and Therapeutic Interventions. Child Dev. Perspect..

[3] Purewal R., Christley R., Kordas K., Joinson C., Meints K., Gee N., Westgarth C. (2017). Companion Animals and Child/Adolescent Development: A Systematic Review of the Evidence. Int. J. Environ. Res. Public. Health.

[4] Tomlinson C.A., Matijczak A., McDonald S.E., Gee N.R., Halpern-Fisher B. (2021). The role of human-animal interaction in child and adolescent development. The Encyclopedia of Child and Adolescent Health.

[5] (1998). American Veterinary Medical Association Statement from the Committee on the Human-Animal Bond. J. Am. Vet. Med. Assoc..

[6] Laing M., Maylea C. (2018). “They Burn Brightly, But Only for a Short Time”: The Role of Social Workers in Companion Animal Grief and Loss. Anthrozoös.

[7] Risley-Curtiss C. (2010). Social Work Practitioners and the Human-Companion Animal Bond: A National Study. Soc. Work.

[8] Morley C., Fook J. (2005). The Importance of Pet Loss and Some Implications for Services. Mortality.

[9] McCune S., McCardle P., Griffin J.A., Esposito L., Hurley K., Bures R., Kruger K.A. (2020). Editorial: Human-Animal Interaction (HAI) Research: A Decade of Progress. Front. Vet. Sci..

[10] Meints K., Brelsford V., Dimolareva M., Gee N. Dog-Assisted Interventions with Children in Mainstream and Special Educational Needs Schools—What Works? In Proceedings of the International Society for Anthrozoology Annual Conference, Liverpool, UK, 5 September 2020.

[11] Rew L. (2000). Friends and Pets as Companions: Strategies for Coping with Loneliness among Homeless Youth. J. Child Adolesc. Psychiatr. Nurs..

[12] Rhoades H., Winetrobe H., Rice E. (2015). Pet Ownership among Homeless Youth: Associations with Mental Health, Service Utilization and Housing Status. Child Psychiatry Hum. Dev..

[13] Triebenbacher S.L., Wilson C.C., Turner D.C. (1998). The relationship between attachment to companion animals and self-esteem: A developmental perspective. Companion Animals in Human Health.

[14] Mathers M., Canterford L., Olds T., Waters E., Wake M. (2010). Pet Ownership and Adolescent Health: Cross-Sectional Population Study. J. Paediatr. Child Health.

[15] Matijczak A., McDonald S.E., O’Connor K.E., George N., Tomlinson C.A., Murphy J.L., Ascione F.R., Williams J.H. (2020). Do Animal Cruelty Exposure and Positive Engagement with Pets Moderate Associations between Children’s Exposure to Intimate Partner Violence and Externalizing Behavior Problems?. Child Adolesc. Soc. Work J..

[16] Hartwig E., Signal T. (2020). Attachment to Companion Animals and Loneliness in Australian Adolescents. Aust. J. Psychol..

[17] Paul E.S., Serpell J.A. (1996). Obtaining a New Pet Dog: Effects on Middle Childhood Children and Their Families. Appl. Anim. Behav. Sci..

[18] Crawford K.M., Zhu Y., Davis K.A., Ernst S., Jacobsson K., Nishimi K., Smith A.D.A.C., Dunn E.C. (2020). The Mental Health Effects of Pet Death during Childhood: Is It Better to Have Loved and Lost than Never to Have Loved at All?. Eur. Child Adolesc. Psychiatry.

[19] Barker S.B., Schubert C.M., Barker R.T., Kuo S.I.C., Kendler K.S., Dick D.M. (2020). The Relationship between Pet Ownership, Social Support, and Internalizing Symptoms in Students from the First to Fourth Year of College. Appl. Dev. Sci..

[20] Matijczak A., McDonald S.E., Tomlinson C.A., Murphy J.L., O’Connor K. (2021). The Moderating Effect of Comfort from Companion Animals and Social Support on the Relationship between Microaggressions and Mental Health in LGBTQ+ Emerging Adults. Behav. Sci..

[21] Carlisle G.K., Johnson R.A., Wang Z., Brosi T.C., Rife E.M., Hutchison A. (2020). Exploring Human–Companion Animal Interaction in Families of Children with Autism. J. Autism Dev. Disord..

[22] Fecteau S.-M., Boivin L., Trudel M., Corbett B.A., Harrell F.E., Viau R., Champagne N., Picard F. (2017). Parenting Stress and Salivary Cortisol in Parents of Children with Autism Spectrum Disorder: Longitudinal Variations in the Context of a Service Dog’s Presence in the Family. Biol. Psychol..

[23] Hall S.S., Wright H.F., Hames A., Mills D.S., Team P. (2016). The Long-Term Benefits of Dog Ownership in Families with Children with Autism. J. Vet. Behav..

[24] Hall S.S., Gee N.R., Mills D.S. (2016). Reading to Dogs: A Systematic Review of the Literature. PLoS ONE.

[25] McCullough A., Ruehrdanz A., Jenkins M.A., Gilmer M.J., Olson J., Pawar A., Holley L., Sierra-Rivera S., Linder D.E., Pichette D. (2018). Measuring the Effects of an Animal-Assisted Intervention for Pediatric Oncology Patients and Their Parents: A Multisite Randomized Controlled Trial. J. Pediatr. Oncol. Nurs..

[26] Wright H.F., Hall S., Hames A., Hardiman J., Mills R., Mills D.S., PAWS Team (2015). Acquiring a Pet Dog Significantly Reduces Stress of Primary Carers for Children with Autism Spectrum Disorder: A Prospective Case Control Study. J. Autism Dev. Disord..

[27] Applebaum J.W., MacLean E.L., McDonald S.E. (2021). Love, Fear, and the Human-Animal Bond: On Adversity and Multispecies Relationships. Compr. Psychoneuroendocrinol..

[28] Applebaum J.W., Tomlinson C.A., Matijczak A., McDonald S.E., Zsembik B.A. (2020). The Concerns, Difficulties, and Stressors of Caring for Pets during COVID-19: Results from a Large Survey of U.S. Pet Owners. Animals.

[29] McDonald S.E., O’Connor K., Matijczak A., Murphy J., Applebaum J.W., Tomlinson C.A., Wike T.L., Kattari S.K. (2021). Victimization and Psychological Wellbeing among Sexual and Gender Minority Emerging Adults: Testing the Moderating Role of Emotional Comfort from Companion Animals. J. Soc. Soc. Work Res..

[30] Mueller M.K., King E.K., Callina K., Dowling-Guyer S., McCobb E. (2021). Demographic and Contextual Factors as Moderators of the Relationship between Pet Ownership and Health. Health Psychol. Behav. Med..

[31] Tomlinson C.A., Murphy J.L., Williams J.M., Hawkins R.D., Matijczak A., Applebaum J.W., McDonald S.E. (2021). Testing the Moderating Role of Victimization and Microaggressions on the Relationship between Human-Animal Interaction and Psychological Adjustment among LGBTQ+ Emerging Adults. Hum. Anim. Interact. Bull..

[32] Adane A.A., Bailey H.D., Morgan V.A., Galbally M., Farrant B.M., Marriott R., White S.W., Shepherd C.C.J. (2021). The Impact of Maternal Prenatal Mental Health Disorders on Stillbirth and Infant Mortality: A Systematic Review and Meta-Analysis. Arch. Womens Ment. Health.

[33] Hazell Raine K., Nath S., Howard L.M., Cockshaw W., Boyce P., Sawyer E., Thorpe K. (2020). Associations between Prenatal Maternal Mental Health Indices and Mother–Infant Relationship Quality 6 to 18 Months’ Postpartum: A Systematic Review. Infant Ment. Health J..

[34] Kingston D., Tough S., Whitfield H. (2012). Prenatal and Postpartum Maternal Psychological Distress and Infant Development: A Systematic Review. Child Psychiatry Hum. Dev..

[35] Kinser P.A., Thacker L.R., Lapato D., Wagner S., Roberson-Nay R., Jobe-Shields L., Amstadter A., York T.P. (2018). Depressive Symptom Prevalence and Predictors in the First Half of Pregnancy. J. Womens Health.

[36] Shonkoff J.P., Boyce W.T., Levitt P., Martinez F.D., McEwen B. (2021). Leveraging the Biology of Adversity and Resilience to Transform Pediatric Practice. Pediatrics.

[37] Howard L.M., Khalifeh H. (2020). Perinatal Mental Health: A Review of Progress and Challenges. World Psychiatry.

[38] Kinser P.A., Lyon D.E. (2014). A Conceptual Framework of Stress Vulnerability, Depression, and Health Outcomes in Women: Potential Uses in Research on Complementary Therapies for Depression. Brain Behav..

[39] Luca D.L., Margiotta C., Staatz C., Garlow E., Christensen A., Zivin K. (2020). Financial Toll of Untreated Perinatal Mood and Anxiety Disorders Among 2017 Births in the United States. Am. J. Public Health.

[40] Leach L.S., Poyser C., Cooklin A.R., Giallo R. (2016). Prevalence and Course of Anxiety Disorders (and Symptom Levels) in Men across the Perinatal Period: A Systematic Review. J. Affect. Disord..

[41] Shorey S., Chan V. (2020). Paternal Mental Health during the Perinatal Period: A Qualitative Systematic Review. J. Adv. Nurs..

[42] McKee K., Admon L.K., Winkelman T.N.A., Muzik M., Hall S., Dalton V.K., Zivin K. (2020). Perinatal Mood and Anxiety Disorders, Serious Mental Illness, and Delivery-Related Health Outcomes, United States, 2006. BMC Womens Health.

[43] Zeng L.-N., Chen L.-G., Yang C.-M., Zeng L.-P., Zhang L.-Y., Peng T.-M. (2021). Mental Health Care for Pregnant Women in the COVID-19 Outbreak Is Urgently Needed. Women Birth.

[44] Kinser P.A., Jallo N., Amstadter A., Thacker L., Jones E., Moyer S., Rider A.M., Karjane N., Salisbury A. (2021). Depression, Anxiety, Resilience, and Coping: The Experience of Pregnant and New Mothers during the First Few Months of the COVID-19 Pandemic. J. Womens Health.

[45] Lau Y., Wong D.F.K., Wang Y., Kwong D.H.K., Wang Y. (2014). The Roles of Social Support in Helping Chinese Women with Antenatal Depressive and Anxiety Symptoms Cope With Perceived Stress. Arch. Psychiatr. Nurs..

[46] Roos A., Faure S., Lochner C., Vythilingum B., Stein D.J. (2013). Predictors of Distress and Anxiety during Pregnancy. Afr. J. Psychiatry.

[47] Cox T. (1987). Stress, Coping and Problem Solving. Work Stress.

[48] Fink G. (2016). Stress, Definitions, Mechanisms, and Effects Outlined. Stress: Concepts, Cognition, Emotion, and Behavior.

[49] Lazarus R.S. (1966). Psychological Stress and the Coping Process.

[50] Lazarus R.S., Folkman S. (1984). Stress, Appraisal, and Coping.

[51] Chovatiya R., Medzhitov R. (2014). Stress, Inflammation, and Defense of Homeostasis. Mol. Cell.

[52] Monaghan P., Spencer K.A. (2014). Stress and Life History. Curr. Biol..

[53] Field T., Diego M., Hernandez-Reif M., Deeds O., Holder V., Schanberg S., Kuhn C. (2009). Depressed Pregnant Black Women Have a Greater Incidence of Prematurity and Low Birthweight Outcomes. Infant Behav. Dev..

[54] Gavin A.R., Melville J.L., Rue T., Guo Y., Dina K.T., Katon W.J. (2011). Racial Differences in the Prevalence of Antenatal Depression. Gen. Hosp. Psychiatry.

[55] Kim H.G., Kuendig J., Prasad K., Sexter A. (2020). Exposure to Racism and Other Adverse Childhood Experiences Among Perinatal Women with Moderate to Severe Mental Illness. Community Ment. Health J..

[56] Scott K.A., Britton L., McLemore M.R. (2019). The Ethics of Perinatal Care for Black Women: Dismantling the Structural Racism in “Mother Blame” Narratives. J. Perinat. Neonatal Nurs..

[57] Miyake Y., Tanaka K., Sasaki S., Hirota Y. (2011). Employment, Income, and Education and Risk of Postpartum Depression: The Osaka Maternal and Child Health Study. J. Affect. Disord..

[58] Tuten M. (2003). Comparing Homeless and Domiciled Pregnant Substance Dependent Women on Psychosocial Characteristics and Treatment Outcomes. Drug Alcohol Depend..

[59] Yamamoto N., Abe Y., Arima K., Nishimura T., Akahoshi E., Oishi K., Aoyagi K. (2014). Mental Health Problems and Influencing Factors in Japanese Women 4 Months after Delivery. J. Physiol. Anthropol..

[60] Herbell K., Zauszniewski J.A. (2019). Stress Experiences and Mental Health of Pregnant Women: The Mediating Role of Social Support. Issues Ment. Health Nurs..

[61] Aktan N.M. (2012). Social Support and Anxiety in Pregnant and Postpartum Women: A Secondary Analysis. Clin. Nurs. Res..

[62] Corrigan C.P., Kwasky A.N., Groh C.J. (2015). Social Support, Postpartum Depression, and Professional Assistance: A Survey of Mothers in the Midwestern United States. J. Perinat. Educ..

[63] Negron R., Martin A., Almog M., Balbierz A., Howell E.A. (2013). Social Support During the Postpartum Period: Mothers’ Views on Needs, Expectations, and Mobilization of Support. Matern. Child Health J..

[64] Islam J., Broidy L., Baird K., Mazerolle P. (2017). Intimate Partner Violence around the Time of Pregnancy and Postpartum Depression: The Experience of Women of Bangladesh. PLoS ONE.

[65] Khajehei M., Doherty M. (2018). Women’s Experience of Their Sexual Function during Pregnancy and after Childbirth: A Qualitative Survey. Br. J. Midwifery.

[66] Zhang Y., Zou S., Cao Y., Zhang Y. (2012). Relationship between Domestic Violence and Postnatal Depression among Pregnant Chinese Women. Int. J. Gynecol. Obstet..

[67] Huschke S., Murphy-Tighe S., Barry M. (2020). Perinatal Mental Health in Ireland: A Scoping Review. Midwifery.

[68] Johnstone S.J., Boyce P.M., Hickey A.R., Morris-Yates A.D., Harris M.G. (2001). Obstetric Risk Factors for Postnatal Depression in Urban and Rural Community Samples. Aust. N. Z. J. Psychiatry.

[69] Mezey G., Bacchus L., Bewley S., White S. (2005). Domestic Violence, Lifetime Trauma and Psychological Health of Childbearing Women. BJOG Int. J. Obstet. Gynaecol..

[70] Bauer G.R. (2014). Incorporating Intersectionality Theory into Population Health Research Methodology: Challenges and the Potential to Advance Health Equity. Soc. Sci. Med..

[71] Norhayati M.N., Nik Hazlina N.H., Asrenee A.R., Wan Emilin W.M.A. (2015). Magnitude and Risk Factors for Postpartum Symptoms: A Literature Review. J. Affect. Disord..

[72] Howell E.A., Mora P., Leventhal H. (2006). Correlates of Early Postpartum Depressive Symptoms. Matern. Child Health J..

[73] Pooler J., Perry D.F., Ghandour R.M. (2013). Prevalence and Risk Factors for Postpartum Depressive Symptoms Among Women Enrolled in WIC. Matern. Child Health J..

[74] Lara-Cinisomo S., Clark C.T., Wood J. (2018). Increasing Diagnosis and Treatment of Perinatal Depression in Latinas and African American Women: Addressing Stigma Is Not Enough. Womens Health Issues.

[75] Bodnar-Deren S., Benn E.K.T., Balbierz A., Howell E.A. (2017). Stigma and Postpartum Depression Treatment Acceptability Among Black and White Women in the First Six-Months Postpartum. Matern. Child Health J..

[76] Corwin E.J., Ferranti E.P. (2016). Integration of Biomarkers to Advance Precision Nursing Interventions for Family Research across the Life Span. Nurs. Outlook.

[77] Berlin L.J., Martoccio T.L., Bryce C.I., Jones Harden B. (2019). Improving Infants’ Stress-Induced Cortisol Regulation through Attachment-Based Intervention: A Randomized Controlled Trial. Psychoneuroendocrinology.

[78] Hagenaars M.A., Oitzl M., Roelofs K. (2014). Updating Freeze: Aligning Animal and Human Research. Neurosci. Biobehav. Rev..

[79] Corwin E.J., Guo Y., Pajer K., Lowe N., McCarthy D., Schmiege S., Weber M., Pace T., Stafford B. (2013). Immune Dysregulation and Glucocorticoid Resistance in Minority and Low Income Pregnant Women. Psychoneuroendocrinology.

[80] Corwin E.J., Pajer K., Paul S., Lowe N., Weber M., McCarthy D.O. (2015). Bidirectional Psychoneuroimmune Interactions in the Early Postpartum Period Influence Risk of Postpartum Depression. Brain Behav. Immun..

[81] Gunnar M.R., Talge N.M., Herrera A. (2009). Stressor Paradigms in Developmental Studies: What Does and Does Not Work to Produce Mean Increases in Salivary Cortisol. Psychoneuroendocrinology.

[82] Porges S.W. (2011). The Polyvagal Theory: Neurophysiological Foundations of Emotions, Attachment, Communication and Self-Regulation.

[83] Seth S., Lewis A.J., Galbally M. (2016). Perinatal Maternal Depression and Cortisol Function in Pregnancy and the Postpartum Period: A Systematic Literature Review. BMC Pregnancy Childbirth.

[84] Cox E.Q., Stuebe A., Pearson B., Grewen K., Rubinow D., Meltzer-Brody S. (2015). Oxytocin and HPA Stress Axis Reactivity in Postpartum Women. Psychoneuroendocrinology.

[85] Leff-Gelman P., Mancilla-Herrera I., Flores-Ramos M., Cruz-Fuentes C., Reyes-Grajeda J.P., del Pilar García-Cuétara M., Bugnot-Pérez M.D., Pulido-Ascencio D.E. (2016). The Immune System and the Role of Inflammation in Perinatal Depression. Neurosci. Bull..

[86] Stuebe A.M., Grewen K., Meltzer-Brody S. (2013). Association Between Maternal Mood and Oxytocin Response to Breastfeeding. J. Womens Health.

[87] Eapen V., Dadds M., Barnett B., Kohlhoff J., Khan F., Radom N., Silove D.M. (2014). Separation Anxiety, Attachment and Inter-Personal Representations: Disentangling the Role of Oxytocin in the Perinatal Period. PLoS ONE.

[88] Kohlhoff J., Eapen V., Dadds M., Khan F., Silove D., Barnett B. (2017). Oxytocin in the Postnatal Period: Associations with Attachment and Maternal Caregiving. Compr. Psychiatry.

[89] Levine A., Zagoory-Sharon O., Feldman R., Weller A. (2007). Oxytocin during Pregnancy and Early Postpartum: Individual Patterns and Maternal–Fetal Attachment. Peptides.

[90] Uvnäs Moberg K., Ekström-Bergström A., Buckley S., Massarotti C., Pajalic Z., Luegmair K., Kotlowska A., Lengler L., Olza I., Grylka-Baeschlin S. (2020). Maternal Plasma Levels of Oxytocin during Breastfeeding—A Systematic Review. PLoS ONE.

[91] Hantsoo L., Jašarević E., Criniti S., McGeehan B., Tanes C., Sammel M.D., Elovitz M.A., Compher C., Wu G., Epperson C.N. (2019). Childhood Adversity Impact on Gut Microbiota and Inflammatory Response to Stress during Pregnancy. Brain Behav. Immun..

[92] Mitchell A.M., Christian L.M. (2019). Repetitive Negative Thinking, Meaning in Life, and Serum Cytokine Levels in Pregnant Women: Varying Associations by Socioeconomic Status. J. Behav. Med..

[93] Leff Gelman P., Mancilla-Herrera I., Flores-Ramos M., Saravia Takashima M.F., Cruz Coronel F.M., Cruz Fuentes C., Pérez Molina A., Hernández-Ruiz J., Silva-Aguilera F.S., Farfan-Labonne B. (2019). The Cytokine Profile of Women with Severe Anxiety and Depression during Pregnancy. BMC Psychiatry.

[94] Karlsson L., Nousiainen N., Scheinin N.M., Maksimow M., Salmi M., Lehto S.M., Tolvanen M., Lukkarinen H., Karlsson H. (2017). Cytokine Profile and Maternal Depression and Anxiety Symptoms in Mid-Pregnancy—the FinnBrain Birth Cohort Study. Arch. Womens Ment. Health.

[95] Osborne L.M., Yenokyan G., Fei K., Kraus T., Moran T., Monk C., Sperling R. (2019). Innate Immune Activation and Depressive and Anxious Symptoms across the Peripartum: An Exploratory Study. Psychoneuroendocrinology.

[96] Del Giudice M., Ellis B.J., Shirtcliff E.A. (2011). The Adaptive Calibration Model of Stress Responsivity. Neurosci. Biobehav. Rev..

[97] Davis E.P., Hankin B.L., Swales D.A., Hoffman M.C. (2018). An Experimental Test of the Fetal Programming Hypothesis: Can We Reduce Child Ontogenetic Vulnerability to Psychopathology by Decreasing Maternal Depression?. Dev. Psychopathol..

[98] Kapoor A., Petropoulos S., Matthews S.G. (2008). Fetal Programming of Hypothalamic–Pituitary–Adrenal (HPA) Axis Function and Behavior by Synthetic Glucocorticoids. Brain Res. Rev..

[99] Park M., Brain U., Grunau R.E., Diamond A., Oberlander T.F. (2018). Maternal Depression Trajectories from Pregnancy to 3 Years Postpartum Are Associated with Children’s Behavior and Executive Functions at 3 and 6 Years. Arch. Womens Ment. Health.

[100] Sandman C.A., Class Q.A., Glynn L.M., Davis E.P. (2016). Neurobehavioral disorders and developmental origins of health and disease. The Epigenome and Developmental Origins of Health and Disease.

[101] Field T., Diego M., Dieter J., Hernandez-Reif M., Schanberg S., Kuhn C., Yando R., Bendell D. (2004). Prenatal Depression Effects on the Fetus and the Newborn. Infant Behav. Dev..

[102] Gonzalez M.Z., Wroblewski K.L., Allen J.P., Coan J.A., Connelly J.J. (2021). *OXTR* DNA Methylation Moderates the Developmental Calibration of Neural Reward Sensitivity. Dev. Psychobiol..

[103] Unternaehrer E., Bolten M., Nast I., Staehli S., Meyer A.H., Dempster E., Hellhammer D.H., Lieb R., Meinlschmidt G. (2016). Maternal Adversities during Pregnancy and Cord Blood Oxytocin Receptor (*OXTR*) DNA Methylation. Soc. Cogn. Affect. Neurosci..

[104] Lawler J.M., Bocknek E.L., McGinnis E.W., Martinez-Torteya C., Rosenblum K.L., Muzik M. (2019). Maternal Postpartum Depression Increases Vulnerability for Toddler Behavior Problems through Infant Cortisol Reactivity. Infancy.

[105] Howland M.A., Sandman C.A., Davis E.P., Stern H.S., Phelan M., Baram T.Z., Glynn L.M. (2021). Prenatal Maternal Mood Entropy Is Associated with Child Neurodevelopment. Emotion.

[106] Goodman S.H., Rouse M.H., Connell A.M., Broth M.R., Hall C.M., Heyward D. (2011). Maternal Depression and Child Psychopathology: A Meta-Analytic Review. Clin. Child Fam. Psychol. Rev..

[107] La Marca-Ghaemmaghami P., La Marca R., Dainese S.M., Haller M., Zimmermann R., Ehlert U. (2013). The Association between Perceived Emotional Support, Maternal Mood, Salivary Cortisol, Salivary Cortisone, and the Ratio between the Two Compounds in Response to Acute Stress in Second Trimester Pregnant Women. J. Psychosom. Res..

[108] Pulopulos M.M., Baeken C., De Raedt R. (2020). Cortisol Response to Stress: The Role of Expectancy and Anticipatory Stress Regulation. Horm. Behav..

[109] Iob E., Kirschbaum C., Steptoe A. (2018). Positive and Negative Social Support and HPA-Axis Hyperactivity: Evidence from Glucocorticoids in Human Hair. Psychoneuroendocrinology.

[110] Ozbay F., Johnson D.C., Dimoulas E., Morgan C.A., Charney D., Southwick S. (2007). Social Support and Resilience to Stress: From Neurobiology to Clinical Practice. Psychiatry Edgmont Pa Townsh..

[111] Staufenbiel S.M., Koenders M.A., Giltay E.J., Elzinga B.M., Manenschijn L., Hoencamp E., van Rossum E.F.C., Spijker A.T. (2014). Recent Negative Life Events Increase Hair Cortisol Concentrations in Patients with Bipolar Disorder. Stress.

[112] Cruwys T., Dingle G.A., Haslam C., Haslam S.A., Jetten J., Morton T.A. (2013). Social Group Memberships Protect against Future Depression, Alleviate Depression Symptoms and Prevent Depression Relapse. Soc. Sci. Med..

[113] Surkan P.J., Peterson K.E., Hughes M.D., Gottlieb B.R. (2006). The Role of Social Networks and Support in Postpartum Women’s Depression: A Multiethnic Urban Sample. Matern. Child Health J..

[114] Seymour-Smith M., Cruwys T., Haslam S.A., Brodribb W. (2017). Loss of Group Memberships Predicts Depression in Postpartum Mothers. Soc. Psychiatry Psychiatr. Epidemiol..

[115] Elmer T., Stadtfeld C. (2020). Depressive Symptoms Are Associated with Social Isolation in Face-to-Face Interaction Networks. Sci. Rep..

[116] Carter C.S., Porges S.W., Freund L.S., McCune S., Esposito L., Gee N.R., McCardle P. (2016). Neural mechanisms underlying human-animal interaction: An evolutionary perspective. The Social Neuroscience of Human-Animal Interaction.

[117] Applebaum J.W., Peek C.W., Zsembik B.A. (2020). Examining US Pet Ownership Using the General Social Survey. Soc. Sci. J..

[118] McConnell A.R., Paige Lloyd E., Humphrey B.T. (2019). We Are Family: Viewing Pets as Family Members Improves Wellbeing. Anthrozoös.

[119] McDonald S.E., Collins E.A., Maternick A., Nicotera N., Graham-Bermann S., Ascione F.R., Williams J.H. (2019). Intimate Partner Violence Survivors’ Reports of Their Children’s Exposure to Companion Animal Maltreatment: A Qualitative Study. J. Interpers. Violence.

[120] McDonald S.E., Matijczak A., Nicotera N., Applebaum J.W., Kremer L., Natoli G., O’Ryan R., Booth L.J., Murphy J.L., Tomlinson C.A. (2021). “He Was like, My Ride or Die”: Sexual and Gender Minority Emerging Adults’ Perspectives on Living with Pets during the Transition to Adulthood. Emerg. Adulthood.

[121] McNicholas J., Collis G.M. (2001). Children’s Representations of Pets in Their Social Networks. Child Care Health Dev..

[122] Wood L., Martin K., Christian H., Nathan A., Lauritsen C., Houghton S., Kawachi I., McCune S. (2015). The Pet Factor—Companion Animals as a Conduit for Getting to Know People, Friendship Formation and Social Support. PLoS ONE.

[123] Hawkins R.D., McDonald S.E., O’Connor K., Matijczak A., Ascione F.R., Williams J.H. (2019). Exposure to Intimate Partner Violence and Internalizing Symptoms: The Moderating Effects of Positive Relationships with Pets and Animal Cruelty Exposure. Child Abuse Negl..

[124] Riggs D.W., Taylor N., Signal T., Fraser H., Donovan C. (2018). People of Diverse Genders and/or Sexualities and Their Animal Companions: Experiences of Family Violence in a Binational Sample. J. Fam. Issues.

[125] Rosenberg S., Riggs D.W., Taylor N., Fraser H. (2020). ‘Being Together Really Helped’: Australian Transgender and Non-Binary People and Their Animal Companions Living through Violence and Marginalisation. J. Sociol..

[126] McDonald S.E., Murphy J.L., Tomlinson C.A., Matijczak A., Applebaum J.W., Wike T.L., Kattari S.K. (2021). Relations Between Sexual and Gender Minority Stress, Personal Hardiness, and Psychological Stress in Emerging Adulthood: Examining Indirect Effects via Human-Animal Interaction. Youth Soc..

[127] McNicholas J., Gilbey A., Rennie A., Ahmedzai S., Dono J.-A., Ormerod E. (2005). Pet Ownership and Human Health: A Brief Review of Evidence and Issues. BMJ.

[128] Wood L., Giles-Corti B., Bulsara M. (2005). The Pet Connection: Pets as a Conduit for Social Capital?. Soc. Sci. Med..

[129] Gee N.R., Mueller M.K., Curl A.L. (2017). Human–Animal Interaction and Older Adults: An Overview. Front. Psychol..

[130] Graham T.M., Glover T.D. (2014). On the Fence: Dog Parks in the (Un)Leashing of Community and Social Capital. Leis. Sci..

[131] Krause-Parello C.A., Gulick E.E., Basin B. (2019). Loneliness, Depression, and Physical Activity in Older Adults: The Therapeutic Role of Human–Animal Interactions. Anthrozoös.

[132] Wells D.L. (2019). The State of Research on Human–Animal Relations: Implications for Human Health. Anthrozoös.

[133] Wood L., Martin K., Christian H., Houghton S., Kawachi I., Vallesi S., McCune S. (2017). Social Capital and Pet Ownership—A Tale of Four Cities. SSM Popul. Health.

[134] Cohen S., McKay G., Baum A., Taylor S.E., Singer J.E. (1984). Social support, stress and the buffering hypothesis: A theoretical analysis. Handbook of Psychology and Health.

[135] Hagerty B.M., Williams R.A., Coyne J.C., Early M.R. (1996). Sense of Belonging and Indicators of Social and Psychological Functioning. Arch. Psychiatr. Nurs..

[136] Kruse J.A., Hagerty B.M., Byers W.S., Gatien G., Williams R.A. (2014). Considering a Relational Model for Depression in Navy Recruits. Mil. Med..

[137] Lee S., Chung J.E., Park N. (2018). Network Environments and Well-Being: An Examination of Personal Network Structure, Social Capital, and Perceived Social Support. Health Commun..

[138] Ditzen B., Neumann I.D., Bodenmann G., von Dawans B., Turner R.A., Ehlert U., Heinrichs M. (2007). Effects of Different Kinds of Couple Interaction on Cortisol and Heart Rate Responses to Stress in Women. Psychoneuroendocrinology.

[139] Pendry P., Vandagriff J.L., Granger D.A., Taylor M.K. (2020). Salivary studies of the social neuroscience of human-animal interaction. Salivary Bioscience.

[140] Beetz A., Uvnäs-Moberg K., Julius H., Kotrschal K. (2012). Psychosocial and Psychophysiological Effects of Human-Animal Interactions: The Possible Role of Oxytocin. Front. Psychol..

[141] Cardoso C., Kingdon D., Ellenbogen M.A. (2014). A Meta-Analytic Review of the Impact of Intranasal Oxytocin Administration on Cortisol Concentrations during Laboratory Tasks: Moderation by Method and Mental Health. Psychoneuroendocrinology.

[142] Miller S.C., Kennedy C.C., DeVoe D.C., Hickey M., Nelson T., Kogan L. (2009). An Examination of Changes in Oxytocin Levels in Men and Women Before and After Interaction With a Bonded Dog. Anthrozoös.

[143] Nagasawa M., Kikusui T., Onaka T., Ohta M. (2009). Dog’s Gaze at Its Owner Increases Owner’s Urinary Oxytocin during Social Interaction. Horm. Behav..

[144] Christian H.E., Westgarth C., Bauman A., Richards E.A., Rhodes R.E., Evenson K.R., Mayer J.A., Thorpe R.J. (2013). Dog Ownership and Physical Activity: A Review of the Evidence. J. Phys. Act. Health.

[145] Coleman K.J., Rosenberg D.E., Conway T.L., Sallis J.F., Saelens B.E., Frank L.D., Cain K. (2008). Physical Activity, Weight Status, and Neighborhood Characteristics of Dog Walkers. Prev. Med..

[146] Dall P.M., Ellis S.L.H., Ellis B.M., Grant P.M., Colyer A., Gee N.R., Granat M.H., Mills D.S. (2017). The Influence of Dog Ownership on Objective Measures of Free-Living Physical Activity and Sedentary Behaviour in Community-Dwelling Older Adults: A Longitudinal Case-Controlled Study. BMC Public Health.

[147] Levine G.N., Allen K., Braun L.T., Christian H.E., Friedmann E., Taubert K.A., Thomas S.A., Wells D.L., Lange R.A. (2013). Pet Ownership and Cardiovascular Risk: A Scientific Statement From the American Heart Association. Circulation.

[148] McConnell A.R., Brown C.M., Shoda T.M., Stayton L.E., Martin C.E. (2011). Friends with Benefits: On the Positive Consequences of Pet Ownership. J. Personal. Soc. Psychol..

[149] Potter K., Teng J.E., Masteller B., Rajala C., Balzer L.B. (2019). Examining How Dog ‘Acquisition’ Affects Physical Activity and Psychosocial Well-Being: Findings from the BuddyStudy Pilot Trial. Animals.

[150] Schulz C., König H.-H., Hajek A. (2020). Differences in Self-Esteem between Cat Owners, Dog Owners, and Individuals without Pets. Front. Vet. Sci..

[151] Van Houtte B.A., Jarvis P.A. (1995). The Role of Pets in Preadolescent Psychosocial Development. J. Appl. Dev. Psychol..

[152] Hinic K., Kowalski M.O., Holtzman K., Mobus K. (2019). The Effect of a Pet Therapy and Comparison Intervention on Anxiety in Hospitalized Children. J. Pediatr. Nurs..

[153] Lynch C.E., Magann E.F., Barringer S.N., Ounpraseuth S.T., Eastham D.G., Lewis S.D., Stowe Z.N. (2014). Pet Therapy Program for Antepartum High-Risk Pregnancies: A Pilot Study. J. Perinatol..

[154] Lem M., Coe J.B., Haley D.B., Stone E., O’Grady W. (2016). The Protective Association between Pet Ownership and Depression among Street-Involved Youth: A Cross-Sectional Study. Anthrozoös.

[155] Liu S., Powell L., Chia D., Russ T.C., McGreevy P.D., Bauman A.E., Edwards K.M., Stamatakis E. (2019). Is Dog Ownership Associated with Mental Health? A Population Study of 68,362 Adults Living in England. Anthrozoös.

[156] Souter M.A., Miller M.D. (2007). Do Animal-Assisted Activities Effectively Treat Depression? A Meta-Analysis. Anthrozoös.

[157] Colombo G., Buono M.D., Smania K., Raviola R., De Leo D. (2006). Pet Therapy and Institutionalized Elderly: A Study on 144 Cognitively Unimpaired Subjects. Arch. Gerontol. Geriatr..

[158] Jessen J., Cardiello F., Baun M.M. (1996). Avian Companionship in Alleviation of Depression, Loneliness, and Low Morale of Older Adults in Skilled Rehabilitation Units. Psychol. Rep..

[159] Barker S.B., Pandurangi A.K., Best A.M. (2003). Effects of Animal-Assisted Therapy on Patients’ Anxiety, Fear, and Depression before ECT. J. ECT.

[160] Braungart-Rieker J.M., Garwood M.M., Powers B.P., Wang X. (2001). Parental Sensitivity, Infant Affect, and Affect Regulation: Predictors of Later Attachment. Child Dev..

[161] Brown S.M., Schlueter L.J., Hurwich-Reiss E., Dmitrieva J., Miles E., Watamura S.E. (2020). Parental Buffering in the Context of Poverty: Positive Parenting Behaviors Differentiate Young Children’s Stress Reactivity Profiles. Dev. Psychopathol..

[162] Kok R., Thijssen S., Bakermans-Kranenburg M.J., Jaddoe V.W.V., Verhulst F.C., White T., van IJzendoorn M.H., Tiemeier H. (2015). Normal Variation in Early Parental Sensitivity Predicts Child Structural Brain Development. J. Am. Acad. Child Adolesc. Psychiatry.

[163] Dreschel N.A., Granger D.A., Freund L.S., McCune S., Esposito L., Gee N.R., McCardle P. (2016). Advancing the social neuroscience of human-animal interaction: The role of salivary bioscience. The Social Neuroscience of Human-Animal Interaction.

[164] Powell L., Edwards K.M., Bauman A., Guastella A.J., Drayton B., Stamatakis E., McGreevy P. (2019). Canine Endogenous Oxytocin Responses to Dog-Walking and Affiliative Human–Dog Interactions. Animals.

[165] Rodriguez K.E., Guérin N.A., Gabriels R.L., Serpell J.A., Schreiner P.J., O’Haire M.E. (2018). The State of Assessment in Human-Animal Interaction Research. Hum. Anim. Interact. Bull..

[166] Beetz A., Bales K., Freund L.S., McCune S., Esposito L., Gee N.R., McCardle P. (2016). Affiliation in human-animal interaction. The Social Neuroscience of Human-Animal Interaction.

[167] MacLean E.L., Gesquiere L.R., Gee N.R., Levy K., Martin W.L., Carter C.S. (2017). Effects of Affiliative Human–Animal Interaction on Dog Salivary and Plasma Oxytocin and Vasopressin. Front. Psychol..

[168] Demakis G.J., McAdams D. (1994). Personality, Social Support and Well-Being among First Year College Students. Coll. Stud. J..

[169] Nagasawa M., Mitsui S., En S., Ohtani N., Ohta M., Sakuma Y., Onaka T., Mogi K., Kikusui T. (2015). Oxytocin-Gaze Positive Loop and the Coevolution of Human-Dog Bonds. Science.

[170] Jenkins J.L. (1986). Physiological Effects of Petting a Companion Animal. Psychol. Rep..

[171] Vormbrock J.K., Grossberg J.M. (1988). Cardiovascular Effects of Human-Pet Dog Interactions. J. Behav. Med..

[172] Demello L.R. (1999). The Effect of the Presence of a Companion-Animal on Physiological Changes Following the Termination of Cognitive Stressors. Psychol. Health.

[173] Handlin L., Hydbring-Sandberg E., Nilsson A., Ejdebäck M., Jansson A., Uvnäs-Moberg K. (2011). Short-Term Interaction between Dogs and Their Owners: Effects on Oxytocin, Cortisol, Insulin and Heart Rate—An Exploratory Study. Anthrozoös.

[174] Charnetski C.J., Riggers S., Brennan F.X. (2004). Effect of Petting a Dog on Immune System Function. Psychol. Rep..

[175] Odendaal J.S.J. (2000). Animal-Assisted Therapy—Magic or Medicine?. J. Psychosom. Res..

[176] Barker S.B., Knisely J.S., McCain N.L., Best A.M. (2005). Measuring Stress and Immune Response in Healthcare Professionals Following Interaction with a Therapy Dog: A Pilot Study. Psychol. Rep..

[177] Pendry P., Vandagriff J.L. (2019). Animal Visitation Program (AVP) Reduces Cortisol Levels of University Students: A Randomized Controlled Trial. AERA Open.

[178] Kertes D.A., Liu J., Hall N.J., Hadad N.A., Wynne C.D.L., Bhatt S.S. (2017). Effect of Pet Dogs on Children’s Perceived Stress and Cortisol Stress Response. Soc. Dev..

[179] Polheber J.P., Matchock R.L. (2014). The Presence of a Dog Attenuates Cortisol and Heart Rate in the Trier Social Stress Test Compared to Human Friends. J. Behav. Med..

[180] Wells D.L. (2004). The Facilitation of Social Interactions by Domestic Dogs. Anthrozoös.

[181] Prothmann A., Ettrich C., Prothmann S. (2009). Preference for, and Responsiveness to, People, Dogs and Objects in Children with Autism. Anthrozoös.

[182] Cardoso C., Ellenbogen M.A., Orlando M.A., Bacon S.L., Joober R. (2013). Intranasal Oxytocin Attenuates the Cortisol Response to Physical Stress: A Dose–Response Study. Psychoneuroendocrinology.

[183] Cochran D.M., Fallon D., Hill M., Frazier J.A. (2013). The Role of Oxytocin in Psychiatric Disorders: A Review of Biological and Therapeutic Research Findings. Harv. Rev. Psychiatry.

[184] Bertsch K., Gamer M., Schmidt B., Schmidinger I., Walther S., Kästel T., Schnell K., Büchel C., Domes G., Herpertz S.C. (2013). Oxytocin and Reduction of Social Threat Hypersensitivity in Women With Borderline Personality Disorder. Am. J. Psychiatry.

[185] Norman G.J., Cacioppo J.T., Morris J.S., Malarkey W.B., Berntson G.G., DeVries A.C. (2011). Oxytocin Increases Autonomic Cardiac Control: Moderation by Loneliness. Biol. Psychol..

[186] Marr C.A., French L., Thompson D., Drum L., Greening G., Mormon J., Henderson I., Hughes C.W. (2000). Animal-Assisted Therapy in Psychiatric Rehabilitation. Anthrozoös.

[187] Yount R., Ritchie E.C., St. Laurent M., Chumley P., Olmert M.D. (2013). The Role of Service Dog Training in the Treatment of Combat-Related PTSD. Psychiatr. Ann..

[188] Kertes D.A., Hall N., Bhatt S.S. (2018). Children’s Relationship With Their Pet Dogs and OXTR Genotype Predict Child–Pet Interaction in an Experimental Setting. Front. Psychol..

[189] Smearman E.L., Yu T., Brody G.H. (2016). Variation in the Oxytocin Receptor Gene Moderates the Protective Effects of a Family-based Prevention Program on Telomere Length. Brain Behav..

[190] Kushner S.C., Herzhoff K., Vrshek-Schallhorn S., Tackett J.L. (2018). Depression in Early Adolescence: Contributions from Relational Aggression and Variation in the Oxytocin Receptor Gene. Aggress. Behav..

[191] Rodrigues S.M., Saslow L.R., Garcia N., John O.P., Keltner D. (2009). Oxytocin Receptor Genetic Variation Relates to Empathy and Stress Reactivity in Humans. Proc. Natl. Acad. Sci. USA.

[192] Krueger F., Parasuraman R., Iyengar V., Thornburg M., Weel J., Lin M., Clarke E., McCabe K., Lipsky R.H. (2012). Oxytocin Receptor Genetic Variation Promotes Human Trust Behavior. Front. Hum. Neurosci..

[193] Saphire-Bernstein S., Way B.M., Kim H.S., Sherman D.K., Taylor S.E. (2011). Oxytocin Receptor Gene (OXTR) Is Related to Psychological Resources. Proc. Natl. Acad. Sci. USA.

[194] Prothmann A., Albrecht K., Dietrich S., Hornfeck U., Stieber S., Ettrich C. (2005). Analysis of Child—Dog Play Behavior in Child Psychiatry. Anthrozoös.

[195] O’Haire M.E., Guérin N.A., Kirkham A.C. (2015). Animal-Assisted Intervention for Trauma: A Systematic Literature Review. Front. Psychol..

[196] O’Haire M.E., Tedeschi P., Jenkins M.A., Braden S.R., Rodriguez K.E., Tedeschi P., Jenkins M.A. (2019). The impact of human-animal interaction in trauma recovery. Transforming Trauma: Resilience and Healing Through our Connections with Animals.

[197] Janssens M., Janssens E., Eshuis J., Lataster J., Simons M., Reijnders J., Jacobs N. (2021). Companion Animals as Buffer against the Impact of Stress on Affect: An Experience Sampling Study. Animals.

[198] Chandler C.K. (2019). Eight Domains of Pet-Owner Wellness: Implications for Counselors and Counselor Training. Clinician’s Guide to Treating Companion Animal Issues.

[199] Beetz A., Schöfmann I., Girgensohn R., Braas R., Ernst C. (2019). Positive Effects of a Short-Term Dog-Assisted Intervention for Soldiers with Post-Traumatic Stress Disorder—A Pilot Study. Front. Vet. Sci..

[200] Crowley-Robinson P., Fenwick D.C., Blackshaw J.K. (1996). A Long-Term Study of Elderly People in Nursing Homes with Visiting and Resident Dogs. Appl. Anim. Behav. Sci..

[201] Connell C.M., Janevic M.R., Solway E., McLaughlin S.J. (2007). Are Pets a Source of Support or Added Burden for Married Couples Facing Dementia?. J. Appl. Gerontol..

[202] Buller K., Ballantyne K.C. (2020). Living with and Loving a Pet with Behavioral Problems: Pet Owners’ Experiences. J. Vet. Behav..

[203] Jakeman M., Oxley J.A., Owczarczak-Garstecka S.C., Westgarth C. (2020). Pet Dog Bites in Children: Management and Prevention. BMJ Paediatr. Open.

[204] Lodge C.J., Lowe A.J., Gurrin L.C., Matheson M.C., Balloch A., Axelrad C., Hill D.J., Hosking C.S., Rodrigues S., Svanes C. (2012). Pets at Birth Do Not Increase Allergic Disease in At-Risk Children. Clin. Exp. Allergy.

[205] Fretzayas A., Kotzia D., Moustaki M. (2013). Controversial Role of Pets in the Development of Atopy in Children. World J. Pediatr..

[206] Ahmed M., Sood A., Gupta J. (2020). Toxoplasmosis in Pregnancy. Eur. J. Obstet. Gynecol. Reprod. Biol..

[207] Kravetz J.D., Federman D.G. (2005). Toxoplasmosis in Pregnancy. Am. J. Med..

[208] Shaevitz M.H., Tullius J.A., Callahan R.T., Fulkerson C.M., Spitznagel M.B. (2020). Early Caregiver Burden in Owners of Pets with Suspected Cancer: Owner Psychosocial Outcomes, Communication Behavior, and Treatment Factors. J. Vet. Intern. Med..

[209] Spitznagel M.B., Jacobson D.M., Cox M.D., Carlson M.D. (2018). Predicting Caregiver Burden in General Veterinary Clients: Contribution of Companion Animal Clinical Signs and Problem Behaviors. Vet. J..

[210] Spitznagel M.B., Mueller M.K., Fraychak T., Hoffman A.M., Carlson M.D. (2019). Validation of an Abbreviated Instrument to Assess Veterinary Client Caregiver Burden. J. Vet. Intern. Med..

[211] Bussolari C.J., Habarth J., Katz R., Phillips S., Carmack B., Packman W. (2018). The Euthanasia Decision-Making Process: A Qualitative Exploration of Bereaved Companion Animal Owners. Bereave. Care.

[212] Habarth J., Bussolari C., Gomez R., Carmack B.J., Ronen R., Field N.P., Packman W. (2017). Continuing Bonds and Psychosocial Functioning in a Recently Bereaved Pet Loss Sample. Anthrozoös.

[213] Hunt M., Padilla Y. (2006). Development of the Pet Bereavement Questionnaire. Anthrozoös.

[214] LaVallee E., Mueller M.K., McCobb E. (2017). A Systematic Review of the Literature Addressing Veterinary Care for Underserved Communities. J. Appl. Anim. Welf. Sci..

[215] Power E.R. (2017). Renting with Pets: A Pathway to Housing Insecurity?. Hous. Stud..

[216] Rose D., McMillian C., Carter O. (2020). Pet-Friendly Rental Housing: Racial and Spatial Inequalities. Space Cult..

[217] Saadeh F.B., Clark M.A., Rogers M.L., Linkletter C.D., Phipps M.G., Padbury J.F., Vivier P.M. (2013). Pregnant and Moving: Understanding Residential Mobility during Pregnancy and in the First Year of Life Using a Prospective Birth Cohort. Matern. Child Health J..

[218] Applebaum J.W., Adams B.L., Eliasson M.N., Zsembik B.A., McDonald S.E. (2020). How Pets Factor into Healthcare Decisions for COVID-19: A One Health Perspective. One Health.

[219] Adams B.L., Applebaum J.W., Eliasson M.N., McDonald S.E., Zsembik B.A. (2021). Child and Pet Care-Planning During COVID-19: Considerations for the Evolving Family Unit. Fam. Relat..

[220] Parfitt Y., Ayers S. (2014). Transition to Parenthood and Mental Health in First-Time Parents. Infant Ment. Health J..

[221] Tham E.K.H., Tan J., Chong Y.-S., Kwek K., Saw S.-M., Teoh O.-H., Goh D.Y.T., Meaney M.J., Broekman B.F.P. (2016). Associations between Poor Subjective Prenatal Sleep Quality and Postnatal Depression and Anxiety Symptoms. J. Affect. Disord..

[222] Andre C.J., Lovallo V., Spencer R.M.C. (2021). The Effects of Bed Sharing on Sleep: From Partners to Pets. Sleep Health.

[223] Mojahed A., Alaidarous N., Kopp M., Pogarell A., Thiel F., Garthus-Niegel S. (2021). Prevalence of Intimate Partner Violence among Intimate Partners during the Perinatal Period: A Narrative Literature Review. Front. Psychiatry.

[224] McDonald S.E., Collins E.A., Nicotera N., Hageman T.O., Ascione F.R., Williams J.H., Graham-Bermann S.A. (2015). Children’s Experiences of Companion Animal Maltreatment in Households Characterized by Intimate Partner Violence. Child Abuse Negl..

[225] Collins E.A., Cody A.M., McDonald S.E., Nicotera N., Ascione F.R., Williams J.H. (2018). A Template Analysis of Intimate Partner Violence Survivors’ Experiences of Animal Maltreatment: Implications for Safety Planning and Intervention. Violence Women.

[226] Monsalve S., Ferreira F., Garcia R. (2017). The Connection between Animal Abuse and Interpersonal Violence: A Review from the Veterinary Perspective. Res. Vet. Sci..

[227] Faver C.A., Strand E.B. (2003). Domestic Violence and Animal Cruelty: Untangling the Web of Abuse. J. Soc. Work Educ..

[228] Moyer S.W., Kinser P.A. (2021). A Comprehensive Conceptual Framework to Guide Clinical Practice and Research About Mental Health during the Perinatal Period. J. Perinat. Neonatal Nurs..

[229] Highet N., Stevenson A.L., Purtell C., Coo S. (2014). Qualitative Insights into Women’s Personal Experiences of Perinatal Depression and Anxiety. Women Birth.

[230] Beetz A.M. (2017). Theories and Possible Processes of Action in Animal Assisted Interventions. Appl. Dev. Sci..

[231] Shoesmith E., Shahab L., Kale D., Mills D.S., Reeve C., Toner P., Santos de Assis L., Ratschen E. (2021). The Influence of Human–Animal Interactions on Mental and Physical Health during the First COVID-19 Lockdown Phase in the U.K.: A Qualitative Exploration. Int. J. Environ. Res. Public. Health.

[232] Carter C.S., Kenkel W.M., MacLean E.L., Wilson S.R., Perkeybile A.M., Yee J.R., Ferris C.F., Nazarloo H.P., Porges S.W., Davis J.M. (2020). Is Oxytocin “Nature’s Medicine”?. Pharmacol. Rev..

[233] Herman J.P., Cullinan W.E. (1997). Neurocircuitry of Stress: Central Control of the Hypothalamo–Pituitary–Adrenocortical Axis. Trends Neurosci..

[234] Milgrom J., Hirshler Y., Reece J., Holt C., Gemmill A.W. (2019). Social Support—A Protective Factor for Depressed Perinatal Women?. Int. J. Environ. Res. Public. Health.

[235] Rallis S., Skouteris H., McCabe M., Milgrom J. (2014). The Transition to Motherhood: Towards a Broader Understanding of Perinatal Distress. Women Birth.

[236] Scorza P., Merz E.C., Spann M., Steinberg E., Feng T., Lee S., Werner E., Peterson B.S., Monk C. (2021). Pregnancy-Specific Stress and Sensitive Caregiving during the Transition to Motherhood in Adolescents. BMC Pregnancy Childbirth.

[237] Louie A.D., Cromer L.D., Berry J.O. (2017). Assessing Parenting Stress: Review of the Use and Interpretation of the Parental Stress Scale. Fam. J..

[238] Nomaguchi K.M., Brown S.L. (2011). Parental Strains and Rewards Among Mothers: The Role of Education. J. Marriage Fam..

[239] Nomaguchi K.M., Milkie M.A. (2003). Costs and Rewards of Children: The Effects of Becoming a Parent on Adults’ Lives. J. Marriage Fam..

[240] Fewtrell M.S. (2005). Randomised, Double Blind Trial of Oxytocin Nasal Spray in Mothers Expressing Breast Milk for Preterm Infants. Arch. Dis. Child. Fetal Neonatal Ed..

[241] Ruis H., Rolland R., Doesburg W., Broeders G., Corbey R. (1981). Oxytocin Enhances Onset of Lactation among Mothers Delivering Prematurely. BMJ.

[242] Kingston D., Kehler H., Austin M.-P., Mughal M.K., Wajid A., Vermeyden L., Benzies K., Brown S., Stuart S., Giallo R. (2018). Trajectories of Maternal Depressive Symptoms during Pregnancy and the First 12 Months Postpartum and Child Externalizing and Internalizing Behavior at Three Years. PLoS ONE.

[243] Mughal M.K., Giallo R., Arnold P., Benzies K., Kehler H., Bright K., Kingston D. (2018). Trajectories of Maternal Stress and Anxiety from Pregnancy to Three Years and Child Development at 3 Years of Age: Findings from the All Our Families (AOF) Pregnancy Cohort. J. Affect. Disord..

[244] Tien J., Lewis G.D., Liu J. (2020). Prenatal Risk Factors for Internalizing and Externalizing Problems in Childhood. World J. Pediatr..

[245] Davis E.P., Glynn L.M., Waffarn F., Sandman C.A. (2011). Prenatal Maternal Stress Programs Infant Stress Regulation: Prenatal Cortisol and Infant Development. J. Child Psychol. Psychiatry.

[246] Werner E., Zhao Y., Evans L., Kinsella M., Kurzius L., Altincatal A., McDonough L., Monk C. (2012). Higher Maternal Prenatal Cortisol and Younger Age Predict Greater Infant Reactivity to Novelty at 4 Months: An Observation-Based Study. Dev. Psychobiol..

[247] Graham A.M., Rasmussen J.M., Rudolph M.D., Heim C.M., Gilmore J.H., Styner M., Potkin S.G., Entringer S., Wadhwa P.D., Fair D.A. (2018). Maternal Systemic Interleukin-6 during Pregnancy Is Associated with Newborn Amygdala Phenotypes and Subsequent Behavior at 2 Years of Age. Biol. Psychiatry.

[248] Rasmussen J.M., Graham A.M., Entringer S., Gilmore J.H., Styner M., Fair D.A., Wadhwa P.D., Buss C. (2019). Maternal Interleukin-6 Concentration during Pregnancy Is Associated with Variation in Frontolimbic White Matter and Cognitive Development in Early Life. NeuroImage.

[249] Bernier A., Carlson S.M., Whipple N. (2010). From External Regulation to Self-Regulation: Early Parenting Precursors of Young Children’s Executive Functioning. Child Dev..

[250] Lunkenheimer E., Busuito A., Brown K.M., Skowron E.A. (2018). Mother-Child Coregulation of Parasympathetic Processes Differs by Maltreatment Severity and Subtype. Child Maltreat..

[251] Skowron E.A., Cipriano-Essel E., Benjamin L.S., Pincus A.L., Van Ryzin M.J. (2013). Cardiac Vagal Tone and Quality of Parenting Show Concurrent and Time-Ordered Associations That Diverge in Abusive, Neglectful, and Non-Maltreating Mothers. Couple Fam. Psychol. Res. Pract..

[252] Kalomiris A.E., Kiel E.J. (2018). Mother-Toddler Cortisol Synchrony Moderates Risk of Early Internalizing Symptoms. Infancy.

[253] Chalmers B. (2017). Family-Centred Perinatal Care: Improving Pregnancy, Birth and Postpartum Care.

[254] Chalmers B., Davis-Floyd R. (2021). Family-centered, evidence-based, psycho-socially sensitive, and culturally respectful perinatal care: Still a futuristic dream!. Birthing Techno-Sapiens: Human-Technology Co-Evolution and the Future of Reproduction.

[255] Franck L.S., O’Brien K. (2019). The Evolution of Family-centered Care: From Supporting Parent-delivered Interventions to a Model of Family Integrated Care. Birth Defects Res..

[256] Paulson J.F., Bazemore S.D. (2010). Prenatal and Postpartum Depression in Fathers and Its Association With Maternal Depression: A Meta-Analysis. JAMA.

[257] Paulson J.F., Bazemore S.D., Goodman J.H., Leiferman J.A. (2016). The Course and Interrelationship of Maternal and Paternal Perinatal Depression. Arch. Womens Ment. Health.

[258] Skjøthaug T., Fitzgerald H.E., von Klitzing K., Cabrera N.J., Scarano de Mendonça J., Skjøthaug T. (2020). Antecedents of Fathers’ Stress in Fatherhood. Handbook of Fathers and Child Development.

[259] McCall C.E., Rodriguez K.E., Wadsworth S.M.M., Meis L.A., O’Haire M.E. (2020). “A Part of Our Family”? Effects of Psychiatric Service Dogs on Quality of Life and Relationship Functioning in Military-Connected Couples. Mil. Behav. Health.

[260] Nieforth L.O., Craig E.A., Behmer V.A., MacDermid Wadsworth S., O’Haire M.E. (2021). PTSD Service Dogs Foster Resilience among Veterans and Military Families. Curr. Psychol..

[261] Leahy-Warren P., McCarthy G., Corcoran P. (2012). First-Time Mothers: Social Support, Maternal Parental Self-Efficacy and Postnatal Depression: First-Time Mothers. J. Clin. Nurs..

[262] Carter C.S. (2017). The Oxytocin–Vasopressin Pathway in the Context of Love and Fear. Front. Endocrinol..

